# Phytochemical Investigation and Biological Activities of *Desmodium heterocarpon* Extract as Anti-Tyrosinase: Isolation of Natural Compounds, *In Vitro* and *In Silico* Study

**DOI:** 10.3390/life14111400

**Published:** 2024-10-31

**Authors:** Suthinee Sangkanu, Wanlapa Nuankaew, Thanet Pitakbut, Sukanya Dej-adisai

**Affiliations:** 1 Department of Pharmacognosy and Pharmaceutical Botany, Faculty of Pharmaceutical Sciences, Prince of Songkla University, Hat Yai 90112, Songkhla, Thailand; suthinee.9938@gmail.com (S.S.); wanlapa.nuankaew@gmail.com (W.N.); 2 Pharmaceutical Biology, Department of Biology, Friedrich-Alexander-Universität Erlangen-Nürnberg (FAU), 91058 Erlangen, Germany; thanet.pitakbut@fau.de

**Keywords:** *Desmodium heterocarpon*, tyrosinase inhibitory activity, 2,5-dihydroxybenzoic acid, molecular docking stimulation, antimicrobial activity

## Abstract

Tyrosinase is an important enzyme in the biosynthesis of melanin. Many skin-whitening agents that inhibit tyrosinase activity from natural sources have been identified because they are harmless and non-toxic. In this work, 114 samples of 54 Fabaceae plants were assessed for their anti-tyrosinase activity using a dopachrome method. The results found that *Desmodium heterocarpon* stems and roots demonstrated the highest tyrosinase inhibitory activity at 20 µg/mL (92.50 ± 1.09%), whereas the water extract of *Artocarpus lacucha* and kojic acid demonstrated 87.41 ± 0.61% and 95.71 ± 0.33%, respectively. Six compounds were isolated from this plant, including genistein (**1**); hexadecanoic acid (**2**); salicylic acid (**3**); β-sitosterol-D-glucoside (**4**); 2,3-dihydroxybenzoic acid (**5**); and 2,5-dihydroxybenzoic acid (**6**). Among them, 2,5-dihydroxybenzoic acid demonstrated a potential effect for tyrosinase inhibition with an IC_50_ of 57.38 µg/mL, while standards of kojic acid and the water extract of *A. lacucha* showed 2.46 and 0.15 µg/mL, respectively. 2,3-dihydroxybenzoic acid had a similar structure as 2,5-dihydroxybenzoic acid; however, it was shown to have tyrosinase inhibitory activity, with an IC_50_ of 128.89 µg/mL. Studies using computer simulations confirmed this reservation. The determination of antimicrobial activities found that 2,5-dihydroxybenzoic acid showed the strongest inhibitory activity against *Staphylococcus aureus*, with MIC and MBC of 5 and 5 µg/mL, respectively. In addition, it inhibited MRSA, *S. epidermidis*, *Propionibacterium acnes*, *Escherichia coli*, and *Pseudomonas aeruginosa*, with MIC and MBC of 15–30 and 15–40 µg/mL. It showed potential activities against yeast and filamentous fungi, such as *Candida albicans*, *Microsporum gypseum*, *Trichophyton rubrum*, and *T. mentagrophytes*, with MIC and MFC of 15 µg/mL. So, 2,5-dihydroxybenzoic acid could inhibit tyrosinase activity and microorganisms that cause skin diseases. Therefore, it can be concluded that this plant has advantageous properties that will be investigated and further developed for possible uses, particularly in the cosmetic and pharmaceutical industries.

## 1. Introduction

The pigment found in human skin cells create ranges in colors from the darkest brown to the lightest pinkish white. The melanin pigment is the most important element. Living things such as humans, animals, plants, and microorganisms (fungi and bacteria) contain it in large quantities. It is a distinct and common pigment that is well known for being able to absorb UV sunlight and eliminate reactive oxygen species (ROS), thus assisting in preserving human skin from the harmful impacts of UV radiation [1]. Melanin is a biopolymer pigment that is diverse and resembles polyphenols. The biological and biochemical pathway of melanin biosynthesis is known as melanogenesis. Tyrosinase is a glycoprotein that is abundant in both plants and animals and functions as the main participant in the melanogenesis pathway [2]. Nevertheless, excessive melanin was connected to tyrosinase overexpression, which resulted in hyperpigmentation and melanoma diseases [3]. Therefore, tyrosinase inhibitors have received more attention as ingredients in medications and cosmetics intended to treat or prevent pigmentation disorders [4]. Naturally occurring tyrosinase inhibitors have consequently gained recognition, as they are widely regarded as safe and devoid of negative side effects.

The genus *Desmodium* is a member of the Fabaceae family, which contains about 350 species. The majority of these species are herbs, shrubs, or sub-shrubs. This genus originated in tropical America and is now widely distributed in East Asia, Mexico, and South America. *Desmodium* has been provided with more than 200 secondary metabolites such as flavonoids, alkaloids, steroids, terpenoids, phenols, phenylpropanoids, glycosides, and volatile oils, which have been determined to have biological activities. Different components of this plant were used in traditional medicine to cure a wide range of diseases, including skin disorders, kidney and vesical stones, mumps, cough, fainting, seizures, and pandemic encephalitis B. Indian medicine employed the leaves of *D. gyrans* (L. f.) DC. to aid in the healing of wounds [5]; the root of *D. laburnifolium* (Poir.) DC. was used to treat digestive and dermatological conditions [6]. The leaves or whole plant of *D. adscendens* (Sw.) DC. were used to cure skin conditions in Nicaragua; in Central America, the root of this species was probably also utilized for skin problems [7]. Ecuador employs *D. molliculum* (Kunth) DC., which was used as a wound therapy [8]. In Thai traditional medicine, *D. heterocarpon* (L.) DC. was used to treat edema [9]. According to Chitra and Narmathabai [10], *D. motorium* (Houtt.) Merr was used for diabetes mellitus, menstrual disorders, tuberculosis, sexual impotence, headaches, and boils. Abscesses, psoriasis, and diarrhea were treated with *D. triflorum* (L.) DC. [11,12]. *D. velutinum* (Willd.) DC. stem and leaves were utilized to treat hypertension [9].

In this work, the researcher finds it interesting to investigate the tyrosinase-inhibiting potential of Thai medicinal plants. After screening for anti-tyrosinase activity, the most promising candidate was *D. heterocarpon* (L.) DC. As a result, the essential chemicals from this plant were isolated and identified. We also examined their antimicrobial properties, and a molecular docking investigation verified the activity. Due to the fact that its active compounds have the ability to inhibit tyrosinase activity and control microbial growth on skin, we suspect that *D. heterocarpon* (L.) DC. plant extract is suitable for use in cosmetic formulations or the management of skin infections.

## 2. Materials and Methods

### 2.1. Plant Materials

A total of 114 samples of 54 Fabaceae plants (Supplementary Materials; Table S1) were collected from the following locations in Thailand: Southern Thai Literary Botanical Garden, Songkhla province, Hala-bala Forest, Narathiwat province, Prince of Songkla University, Central Thai Literary Botanical Garden, Ratchaburi province, Surat Thani, and Songkhla province, Thailand. All of the plants were identified by experts from the Department of National Parks’ Office of the Forest Herbarium and Southern Thai Literary Botanical Garden Songkhla. A herbarium specimen of *D. heterocarpon* (L.) DC. var. *heterocarpon* H. Ohashi was deposited at the Department of Pharmacognosy and Pharmaceutical Botany, Faculty of Pharmaceutical Sciences, Prince of Songkla University, with herbarium number SKP 072 04 08 01.

### 2.2. Preparation of Extracts

An extensive variety of plant parts, including the wood, stem, root, leaf, flower, fruit, and seed parts, were sampled. The samples were broken up into small pieces and dried in a hot air oven for two days at 55 °C. The samples were subsequently soaked in ethanol for a period of seven days. The filtrates were collected and concentrated under low pressure while maintaining a temperature below 45 °C using a vacuum rotary evaporator. Crude extracts were obtained and stored at 4 °C before further study.

### 2.3. Isolation and Purification of Active Compounds

*D. heterocarpon* demonstrated significant potential activity in inhibiting tyrosinase. Consequently, this plant was chosen for additional phytochemical analysis to identify the active ingredients. A total of 3.8 kg of dried plant material (stem and root) was crushed and macerated in several types of solvents. The ground plant was macerated for three days in petroleum ether to begin the extraction process, and the filtrates were then evaporated at 40 °C under low pressure. Next, using the same method as for petroleum ether, the residue was macerated with ethyl acetate and ethanol, respectively. The last phase was extracting the water; the residue was constantly boiled in water at 70 °C for six hours. Anti-tyrosinase activity was observed in the extracts of ethyl acetate, ethanol, and water. Therefore, their selection was based on the requirement to separate and identify the active compounds.

Compound **1** was derived from the ethyl acetate and ethanol extract. A total of 15.41 g of ethyl acetate was fractionated by quick column chromatography using 1 L sintered glass filter and silica gel 60 as the stationary phase. Hexane, acetone, chloroform, and methanol were used as the mobile phase. All of the fractions were examined by the TLC technique for grouping a similar chromatographic pattern and combined. Then, all fractions were tested for tyrosinase inhibition activity. Active anti-tyrosinase fractions Q8 and Q9 (2.1 g) were combined as a result of a similar TLC pattern. Q8–Q9 was subjected to classical column chromatography over a silica gel column (40 cm length × 3 cm i.d.) to yield 16 fractions (Q8/9Si1–Q8/9Si16) with a gradient elution of chloroform and methanol (10:0 to 8:2). Finally, the column was eluted by methanol until the solutions were clear. Fraction 11 (Q8/9Si11; 153.5 mg) showed a distinctive yellow spot on TLC and was further purified by column chromatography over silica gel with gradient elution, using mixtures of hexane and ethyl acetate (10:0 to 0:10), given 10 fractions. Fraction number 2 (Q8Q9Si11Si2; 13.8 mg) was chosen for further isolation. It was purified by the classical column chromatographic technique, using a Sephadex LH-20^®^ column (40 cm length × 1 cm i.d.). The separation was carried out by using mixtures of chloroform and methanol (2:8). Fractions number 9 and 10 yielded a pure compound, obtained as yellow needles in an amount of 3.4 mg, which was called compound **1**. Additionally, we isolated compound **1** from the ethanol extract. A total of 279.7 mg of ethanol extract fraction number 5 (QE5) was separated by the classical column chromatographic technique using a silica gel column (45 cm length × 3 cm i.d.). Gradient elution was performed using mixtures of chloroform and ethyl acetate (10:0 to 0:10); then, the column was eluted by chloroform and methanol (10:0 to 1:1) until the solutions were clear, given 25 fractions (QE5Si1–QE5Si25) after combining the same pattern on TLC. Fraction number 16 (QE5Si16; 20 mg) showed a unique yellow spot on TLC and was further isolated by the classical column chromatographic technique using a Sephadex LH-20^®^ gel column (50 cm length × 1.5 cm i.d.). The separation was carried out by using mixtures of chloroform and methanol (3–7), given 13 fractions (QE5Si16Se1–QE5Si16Se13). Fraction number 10 gave a single spot on the TLC plate, obtained as yellow needles amounting to 2.8 mg along with compound **1**.

Compound **2**, fraction Q6 (543.7 mg), was isolated from the ethyl acetate extract using a classic column chromatographic method (a Sephadex LH-20^®^ column with 60 cm length × 3 cm i.d.). Hexane and ethyl acetate combinations (10:0 to 0:10) were used to purify Q6, resulting in 6 fractions (Q6Se1–QQ6Se6). To continue fractionation, fraction number 3 (Q6Se3) was chosen, since it displayed a significant spot on the TLC. A total of 85.1 mg of Q6Se3 was purified using a classic column chromatographic method (a Sephadex LH-20^®^ column with 85 cm length × 2.5 cm i.d). Chloroform and methanol (1:1) mixtures were used for the separation, yielding 5 fractions (Q6Se3Se1–Q6Se3Se5). A white powder that emerged in fraction number 5 (Q6Se3Se5; 9.5 mg) was chosen to be further purified using a classical column chromatographic process (a silica gel column with 42 cm length × 0.5 cm i.d). Gradient elution was used to separate using hexane and ethyl acetate solutions (10:0 to 0:10). Three fractions (Q6Se3Se5Si1–Q6Se3Se5Si3) showed the same pattern on TLC. Therefore, they were combined and identified as compound **2** (5 mg).

Compound **3**, fraction Q7 (1.53 g) of the ethyl acetate extract, was packed on a silica gel column (45 cm length × 3 cm i.d.) and purified by the classical column chromatographic technique. For gradient elution, hexane and acetone (10:0 to 0:10) were utilized. The column was eluted with methanol until the solutions were clear. Ten fractions (Q7Si1–Q7Si10) were obtained by collecting fractions and combining them on TLC in the same manner. Fractions 6 and 7 (Q7Si6/7; 204.5 mg)) showed the crystals, and then they were selected for combination and further separation. It was subjected over a silica gel column (50 cm length × 3.5 cm i.d.) and purified by the classical column chromatographic technique. The separation was carried out with gradient elution, using mixtures of chloroform and methanol (10:0 to 1:1), after combining all the same pattern fractions on TLC, given 6 fractions (Q7Si6/7Si1–Q7Si6/7Si6). To continue fractionation, fraction number 5 (Q7Si6/7Si5; 39.2 mg) was chosen based on its significant spot on TLC. It was purified using a Sephadex LH-20^®^ column (50 cm length × 2.5 cm i.d.) using a classical column chromatographic method. Chloroform and methanol (2:8) mixes were used for the separation, yielding 21 fractions (Q7Si6/7Si5Se1–Q7Si6/7Si5Se21). Due to the white crystals in fraction number 20 (Q7Si6/7Si5Se20; 9.5 mg), it looks nearly to be a pure compound, so it was selected for further purification by the classical short-column chromatographic technique using a silica gel column (25 cm length × 3.5 cm i.d.). Gradient elution using mixtures of chloroform and methanol (10:0 to 0:10) gave 20 fractions (Q7Si6/7Si5Se20Si1–Q7Si6/7Si5Se20Si20), and fraction number 5 was the cleanest compound, obtained as a colorless crystalline compound in an amount of 3.6 mg, identified as compound **3**.

In compound **4**, significant amounts of white powder were observed in fraction number 10 of the ethanol extract (QE10), leading to its selection for recrystallization using methanol and a tiny amount of chloroform to dissolve and eliminate the impurity. Then, 22.9 mg of recrystallized powder QE10 was purified using silica gel column chromatography (30 cm length × 2.5 cm i.d.). The separation was carried out by using mixtures of chloroform and methanol (9:1), given in 12 fractions. The compound obtained as a white powder at fraction number 9 in an amount of 2.6 mg was identified as compound **4**.

For compounds **5** and **6**, 50 g of water extract was separated by liquid–liquid extraction. The crude extract was dissolved in ethanol and water (1:1) mixes, and the resulting mixtures were partitioned with hexane, chloroform, and ethyl acetate to obtain 11.5, 245.4, and 1125.5 mg, respectively. To carry out the remaining separation, the partitioning ethyl acetate extract was chosen. A total of 1125.5 mg of ethyl acetate extract from partitioning (PTE) was chosen to proceed with fractionation using a Sephadex LH-20^®^ column (75 cm length × 1.5 cm i.d.) using a classic column chromatography method. Methanol was used for the separation, and eight fractions (PTESe1–PTESe8) were obtained by combining the same pattern on TLC. Fraction number 4 (PTESe4) exhibited an exceptional black spot, which was chosen for additional purification using a silica gel column (40 cm length × 2 cm i.d.). Chloroform and combinations of chloroform and 1% formic acid were used to separate 83.3 mg of PTESe4, yielding 4 fractions (PTESe4Si1–PTESe4Si4). The two primary compounds from this column are as follows: the purest chemical, fraction number 2, was produced as a white crystal with a weight of 13.1 mg, identified as compound **5**. Another was fraction number 4 (PTESe4Si4) (13.3 mg), which seemed to have some impurities. As a result, it was purified once again using a Sephadex LH-20^®^ column (40 cm length × 1 cm i.d.). Methanol was used to carry out the separation process. The pure chemical was produced as a cream crystal in an amount of 12.3 mg in fractions 5 and 6 (PTESe4Si4Se5/6), identified as compound **6**.

The isolated compounds were dissolved in methanol or chloroform before measuring UV absorption. IR spectra were recorded on an IR spectrometer at wave number 400–4000 cm^−1^. For NMR spectra, the compounds were dissolved in the solvent (analytic grade) as appropriate. The chemical shifts of ^1^H-NMR and ^13^C-NMR were reported in ppm. The physical properties and spectroscopic data were compared with previous reports.

### 2.4. Biological Studies

#### 2.4.1. Anti-Tyrosinase Activity

The tyrosinase inhibitory activity of the sample extracts was assessed using the dopachrome technique, following the protocol described by Dej-adisai et al. [13]. Briefly, the sample was prepared by dissolving in DMSO at concentration of 1 mg/mL, and then diluted to achieve a concentration of 200 µg/mL. Then, 20 μL of sample was added in a 96-well microplate and mixed with phosphate buffer (pH 6.8). A total of 20 µL of mushroom tyrosinase solution (203.3 U/mL) was added and plate was incubated at 25 °C for 10 min. A total of 20 μL of substrate, L-DOPA (0.85 mM), was added to each well. The visible absorption at time 0 was immediately measured at 492 nm. The mixture was then incubated for 20 min at 25 °C. After the reaction was incubated, the concentration of dopachrome was measured again at 492 nm. Equation (1) was utilized to compute the tyrosinase inhibition values.

% Tyrosinase inhibition = {[(A − B) − (C − D)]/(A − B)} × 100(1)

where A is the difference in optical density before and after incubation at 492 nm without a test sample. B is the difference in optical density before and after incubation at 492 nm without a test sample and enzyme. C is the difference in optical density before and after incubation at 492 nm with a test sample. And D is the difference in optical density before and after incubation at 492 nm with a test sample but without enzymes.

The concentration of the pure compound was measured, which was the half maximal inhibitory concentration (IC_50_). DMSO was employed as the negative control, whereas kojic acid was used as a positive control. Furthermore, since the water extract from *Artocarpus lacucha* wood has been demonstrated to successfully reduce mushroom tyrosinase activity [13], it was also used as a positive control.

#### 2.4.2. Determination of Antimicrobial Activity

Four Gram-positive bacteria were used in this study, including *Staphylococcus aureus* (ATTC 25923), *Staphylococcus epidermidis* (TISTR 517), *Propionibacterium acnes* (DMST 14916), and Methicillin-resistant *Staphylococcus aureus* (MRSA 1350II 06). Two Gram-negative bacteria were *Escherichia coli* ATCC35218 and *Pseudomonas aeruginosa* ATCC10145. The bacterial representatives (except *P. acnes*) were cultivated in Mueller–Hinton broth (MHB) at 37 °C for 18–24 h, while *P. acnes* was cultivated in Brain Heart Infusion broth (BHIB) at 37 °C for 72 h. Then, the inoculum of bacteria (10^6^ CFU/mL) was prepared by adjusting the turbidity with 0.85% NaCl in order to measure the optical density length between 0.085 and 1.3 of the spectrophotometers at 625 nm. After that, the inoculum was diluted in appropriated medium in a ratio of 1: 100.

*Candida albicans* (TISTR 5779) was the yeast reference strain, and *Microsporum gypseum*, *Trichophyton rubrum*, and *Trichophyton mentagrophytes* were representative of filamentous fungi. The yeast and filamentous fungi were cultivated in Sabouraud Dextrose Agar (SDA) at 28 °C for 48 h and 7 days, respectively. The yeast cells and conidia were gathered using 0.85% NaCl, and a hemocytometer was used to count the estimated quantity of fungi. Finally, the fungal suspensions were diluted in SDB medium to achieve a final inoculum size of 10^3^ and 10^5^ CFU/mL for mold and yeast forms, respectively.

The minimal inhibitory concentrations (MICs) of pure compounds were determined by the micro-dilution method using the 96-well plates. This research was carried out in compliance with the guidelines of the Clinical and Laboratory Standards Institute (CLSI) [14,15,16] with modifications. A total of 20 µL of diluted compounds were added to the 96-well plates, which included 80 µL of medium and 100 µL of the inoculum to obtain a final concentration of 1–100 µg/mL. The lowest concentration that inhibited microorganism growth completely (the first clear well) was recorded as the MIC. To determine the minimal bactericidal concentration (MBC) and fungicidal concentration (MFC), 10 µL of all positive wells were subcultured on MHA or SDA medium agar and incubated according to the condition. The MBC or MFC were the lowest concentration that resulted in no growth of bacteria and fungi.

Oxacillin was used as a positive control for *S. aureus*, *S. epidermidis*, and *P. acnes*. Vancomycin served as a positive control for MRSA. For *P. aeruginosa* and *E. coli*, norfloxacin was utilized as a positive control. For *C. albicans*, amphotericin B was employed as a positive control. Additionally, ketoconazole was employed as a positive control for *T. rubrum*, *T. mentagrophytes*, and *M. gypseum*.

### 2.5. Molecular Docking

The authors performed the theoretical docking simulation largely following their previous reports [17,18,19]. The following section provides brief and sufficient information to repeat the simulation experiment with an additional procedure using Gnina, molecular docking with a convolutional neural network (CNN) model [20].

#### 2.5.1. Enzyme and Chemical Structure Preparation

The mushroom tyrosinase crystal structure with tropolone as a native ligand (known tyrosinase inhibitor), PDB ID: 2Y9X [21], was downloaded from the RCSB PBD databank (https://www.rcsb.org/, accessed on 30 July 2024). UCSF Chimera version 1.11.2 [22] was used to prepare a proper protein format file for a docking simulation using Autodock Vina [23]. From this point onward, Autodock Vina is called Vina. In contrast, Autodock Tools version 1.5.7 [24] was utilized for rescore docking via Autodock4 [24] and Gnina [20] software version 1.1.

On the other hand, the 2D chemical structures of isolated compounds from *D. heterocarpon* were obtained from the PubChem database (https://pubchem.ncbi.nlm.nih.gov, accessed on 30 July 2024). The Supplementary File (Table S2) provides all chemical IDs of the selected compounds used. The authors used Open Babel software version 3.1.0 [25] to generate and energetically optimize the 3D chemical structures of all obtained compounds via a general Amber force field (GAFF). After obtaining the optimized 3D structures, Open Babel was also used to prepare a file in proper format for the docking simulation. Finally, Autodock Tools version 1.5.7 [24] was applied to prepare the selected docking pose for the rescore docking experiment.

#### 2.5.2. Docking Simulation

In this study, the authors utilized Vina version 1.2.5 [23] to perform a theoretical docking simulation. The docking experimental condition was set as in our previous publications. The docking grid box was designated as x = −10.1, y = −28.7, and z = −43.4 with a size of 18 cubic Å on the tyrosinase structure. All docking parameters were set as default values except exhaustiveness, which was adjusted to 10. Finally, ten docking poses of each compound were collected, but only one pose was selected (also known as the best pose). Two criteria were applied to assist in docking pose selection. First, the compound with a similar chemical structure will also share a similar docking pose. Second, all selected docking poses should align with the chemical structure of tropolone, the native ligand that came with the tyrosinase crystal structure.

#### 2.5.3. Postdocking Analysis and Rescore Docking

The same UCSF Chimera program was used to analyze Vina docking results in 3D and select the best docking pose and molecular alignment. The authors also used the Ligplot program version 2.2.8 to analyze 2D interactions [26].

After that, all selected docking poses were subjected to a rescore docking experiment using Autodock4 [24] and Gnina [20] to recalculate binding energy and the CNN score (only for Gnina) for further docking evaluation. Here, the authors also applied a default value in both software programs used in the rescore docking experiment.

#### 2.5.4. Statistical Analysis

The Pearson correlation function from Microsoft Excel, version 16.90, was used to evaluate a correlation between the simulation and enzyme binding assay [27].

## 3. Results

### 3.1. Screening of Tyrosinase Inhibitory Activities of Thai Medicinal Plant Extracts

The dopachrome method was utilized to assess the tyrosinase inhibitory activity of Thai plant extracts, with 3,4-dihydroxy-L-phenylalanine (L-DOPA) serving as the substrate. Positive controls, *A. lacucha* (water extract) and kojic acid, at a concentration of 20 µg/mL, showed inhibitory levels of 98.82 ± 0.83% and 92.29 ± 1.93%, respectively. The tyrosinase inhibitory activity of 114 extracts of 54 Fabaceae Thai plants is presented in the Supplementary File (Table S3). At 20 µg/mL of extracts, *D. heterocarpon* (root/stem) showed the highest tyrosinase inhibitory effect at 90.96 ± 1.85%. Consequently, *D. heterocarpon* was chosen to carry out further phytochemical research.

### 3.2. Structure Elucidation of Isolated Compounds from D. Heterocarpon Extract

The dry stem and root of *D. heterocarpon* were extracted in a series of organic solvents with increasing polarity (petroleum ether, ethyl acetate, ethanol, and water). The yields of all extracts are displayed in Table 1. All the extracts were determined to inhibit tyrosinase, and the results showed that the ethyl acetate extract had the highest potential effect of anti-tyrosinase activity, followed by ethanol extract and water extract, as shown in Table 1. We subsequently selected them for the further isolation and purification of pure compounds, which could potentially serve as markers for tyrosinase inhibition.

Six compounds were isolated and purified from ethyl acetate and the ethanol and water extracts of *D. heterocarpon* and were identified as genistein (compound **1**), hexadecanoic acid (compound **2**), salicylic acid (compound **3**), β-sitosterol-D-glucoside (compound **4**), 2,3-dihydroxybenzoic acid (compound **5**), and 2,5-dihydroxybenzoic acid (compound **6**). The isolated compounds were determined by physical properties and spectroscopic data.

Ultraviolet spectroscopy (UV) adsorption spectra were obtained on a Spectronic GenesysTM 6 UV–visible spectrophotometer, Thermo Scientific, Thermo Electron Corporation (Department of Pharmacognosy and Pharmaceutical Botany, Faculty of Pharmaceutical Sciences, Prince of Songkla University). The samples were dissolved in methanol or chloroform before measuring UV absorption.

Infrared spectroscopy (IR) spectra were obtained from a Perkin Elmer FT-IR spectrum one spectrometer (Department of Chemistry, Faculty of Pharmaceutical Sciences, Prince of Songkla University) recorded on an IR spectrometer at wave number 400–4000 cm^−1^. The compounds were prepared as potassium bromide (KBr) pellets to determine the spectra.

Nuclear magnetic resonance spectroscopy (NMR) spectra were recorded on a Fourier transform NMR spectrometer, model UNITY INNOVA, Varian (Office of Scientific Instrument and Testing (OSIT), Prince of Songkla University). The samples were dissolved in the solvent (analysis grade) as appropriate. The chemical shifts of ^1^H-NMR and ^13^C-NMR were reported in ppm.

Electron impact mass spectroscopy (EIMS) was measured on a Thermo Finnigan MAT 95 XL mass spectrometer (OSIT, Prince of Songkla University).

**Figure 1:**
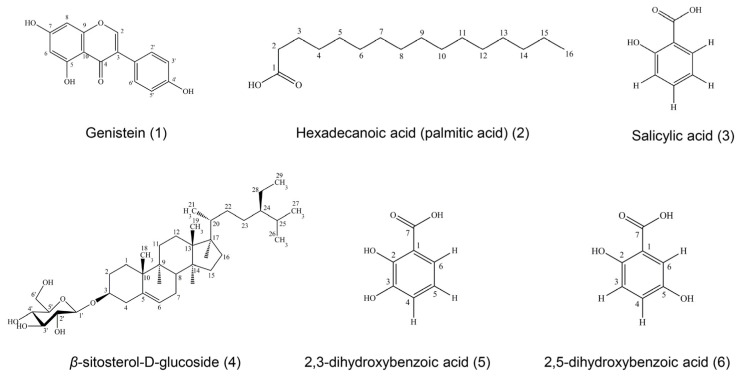
Structures of chemical compounds **1**–**6** detected in *D. heterocarpon* extract.

All data were obtained from UV, IR, NMR, and MS techniques and compared with previous reports (Figure 1).

Compound **1** was obtained as a yellowish needle, soluble in methanol. This compound gave a molecular ion peak at 270 *m*/*z* in the EI mass spectrum, suggesting a tentative molecular formula of C_15_H_10_O_5_. The UV spectrum in methanol showed absorptions at *λ*_max_ (absorbance) 201 (0.508), 210 (0.564), and 262 (0.709) nm. The IR spectrum exhibited maximum absorption bands at 3368.9 (phenolic -OH group), 1615 (carbonyl stretching), and 1045 (O-C stretching) cm^−1^. A comparison of its ^1^H-NMR and ^13^C-NMR spectra with reported data [28] suggested that compound **1** was identical with genistein.

Compound **2** showed white powders, soluble in chloroform. The UV spectrum in chloroform demonstrated absorptions maximal in chloroform at *λ*_max_ (absorbance) 245 (0.271) nm. The IR spectrum exhibited maximum absorption bands at 3400 (phenolic -OH group), 2918 (O-H stretching), and 1700 (C=O stretching) cm^−1^. Compound **2** could be assigned as a long-chain carbon by analyzing its ^1^H-NMR spectrum, as it performed as hexadecanoic acid or palmitic acid. The molecular mass was 256 *m*/*z*, consistent with a molecular formula of C_16_H_32_O_2_.

Compound **3** was obtained as a colorless crystalline substance, soluble in chloroform. The UV spectrum in chloroform showed maximal absorptions at *λ*_max_ (absorbance) 245 (0.429) and 306 (0.435) nm. The IR spectrum exhibited maximum absorption bands at 3200 (O-H stretching) and 1600 (C=O stretching) cm^−1^. Due to the information of ^1^H-NMR, ^1^H-^1^H COSY, and ^13^C-NMR spectra, compound **3** could be assigned as salicylic acid; with a molecular mass of 138 *m*/*z* and a molecular formula of C_7_H_6_O_3_ [29].

Compound **4** was soluble in mixtures of chloroform and methanol, obtained as white powder. The molecular mass was 576 *m*/*z* and the molecular formula was C_35_H_60_O_6_. The UV spectrum in chloroform displayed absorptions at *λ*_max_ (absorbance) 225 (0.277), 249 (0.310), and 327 (0.106) nm. The IR spectrum exhibited maximum absorption bands at 3402 (O-H stretching), 2933 (C-H stretching), 1461 (C=C stretching), and 1023 (C-O stretching) cm^−1^. From ^1^H-NMR and ^13^C-NMR spectra, patterns were similar to *β*-sitosterol-D-glucoside by comparing with those of *β*-sitosterol-D-glucoside [30].

Compound **5** was obtained as a colorless crystalline substance, soluble in methanol. The EI mass spectrum of compound **5** showed a molecular ion peak at 154 *m*/*z*, corresponding to C_7_H_6_O_4_. The UV spectrum in methanol showed absorptions at *λ*_max_ (absorbance) 219 (0.420), 245 (0.218), and 316 (0.127) nm. The IR spectrum exhibited maximal absorption bands at 3371 (O-H stretching), 1675 (C=O stretching), 1474 (C=C stretching), and 1234 (C-O stretching) cm^−1^. Compound **5** could be assigned as the known compound 2,3-dihydroxybenzoic acid or hypogallic acid by analysis of its UV, IR, ^1^H-NMR, ^13^C-NMR, and HMQC spectra properties compared with those of 2,3-dihydroxybenzoic acid [31].

Compound **6** had a cream crystalline appearance, soluble in methanol. This compound presented a molecular ion peak at 154 *m*/*z* in the EI mass spectrum, suggesting a tentative molecular formula of C_7_H_6_O_4_. The UV spectrum in methanol showed absorptions at *λ*_max_ (absorbance) 201 (0.490), 216 (0.693), and 333 (0.167) nm. The IR spectrum exhibited maximum absorption bands at 3374 (O-H stretching), 1662 (C=O stretching), 1443 (C=C stretching), and 1220 (C-O stretching) cm^−1^. Compound **6** could be assigned as the known compound 2,5-dihydroxybenzoic acid or gentisic acid by analysis of its UV, IR, ^1^H-NMR, and ^13^C-NMR spectra properties compared with those of 2,5-dihydroxybenzoic acid [32].

### 3.3. Biological Activities of Isolated Compounds

#### 3.3.1. Investigation of Anti-Tyrosinase Activity of Isolated Compounds

As a result of the experiment on mushroom tyrosinase enzyme, genistein (compound **1**), hexadecanoic acid (compound **2**), *β*-sitosterol-D-glucoside (compound **4**), and 2,3-dihydroxybenzoic acid (compound **5**) showed a weak potency of tyrosinase inhibition. Meanwhile, 2,5-dihydroxybenzoic acid (compound **6**) presented a potential effect for tyrosinase inhibition with an IC_50_ of 57.38 µg/mL (Table 2).

#### 3.3.2. Investigation of Antimicrobial Activity of Isolated Compounds

All of the isolated compounds were tested for antimicrobial activity by a microdilution method except salicylic acid (compound **3**) due to the small amount. Only 2,3-dihydroxybenzoic acid (compound **5**) and 2,5-dihydroxybenzoic acid (compound **6**) showed potential activity on the MIC and MBC/MFC, as shown in Table 3.

### 3.4. Molecular Docking Simulation

The authors performed a molecular docking simulation to support the tyrosinase inhibitory activity *in vitro* mentioned in an earlier study. Before performing the docking simulation, the authors validated the established virtual condition via the re-docking method to ensure the reliability of the simulation. The first extracted a native ligand from a tyrosinase crystal structure and then re-docked the extract native back to its original position under the established virtual condition (Supplementary Materials, Figure S16). The validation result was satisfactory with the root mean square deviation (RMSD) value of 1 Å, passing the acceptance criteria of less than 3 Å. This ensured that the authors’ docking simulation could mimic experimental ligand–tyrosinase binding.

In this simulation, five metabolites isolated from *D. heterocarpon* were analyzed. The first three metabolites are polar compounds such as genistein, 2,3-dihydroxybenzoic acid, and 2,5-dihydroxybenzoic acid, while the remaining two are nonpolar compounds such as palmitic acid and *β*-sitosterol glucoside.

#### 3.4.1. Polar Compound Analysis

**Figure 2:**
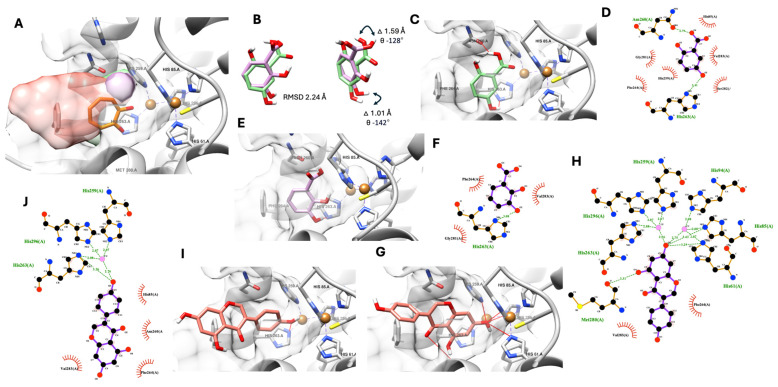
Selected docking pose of three polar metabolites, 2,3-dihydroxybenzoic acid (green), 2,5-dihydroxybenzoic acid (pink), and genistein (red), in the tyrosinase’s active site with tropolone (organ), a native ligand and known competitive inhibitor with two copper ions, shown as a gold-colored sphere (**A**). Molecular alignment and measurement between 2,3-dihydroxybenzoic acid and 2,5-dihydroxybenzoic acid (**B**). The 3D and 2D interaction schemes between tyrosinase’s catalytic pocket and 2,3-dihydroxybenzoic acid (**C**,**D**), 2,5-dihydroxybenzoic acid (**E**,**F**), genistein conformation 1 (**G**,**H**), and genistein conformation 2 (**I**,**J**). The red line indicates predicted hydrogen bonding from a 3D analysis, while the green dashed line indicates hydrogen bonding from a 2D analysis.

The native ligand, which came with the tyrosinase crystal structure, was used to assist each compound’s docking pose selection. Figure 2 shows the selected promising docking pose of three polar compounds of *D. heterocarpon*, see Figure 2A. All three compounds were fitted into the tyrosinase active site, and their polar functional groups faced the copper ions and catalytic residue similar to the native ligand (tropolone, known tyrosinase inhibitor).

The molecular docking simulation suggested proximity in the molecular positioning of 2,3-dihydroxybenzoic acid and 2,5-dihydroxybenzoic acid in the tyrosinase active site with an altered RMSD value of 2.24 Å (Figure 2B), which led to significant different intermolecular interactions (Figure 2C–E). The theoretical docking pose of 2,5-dihydroxybenzoic acid exhibited two possible hydrogen bonds between the carboxylic (-COOH) and position 2 hydroxyl (-OH) groups and Asn260 and His263, two amino acids in the catalytic pocket of tyrosinase, as shown in Figure 2C and Table 4. Six additional hydrophobic interactions were found between 2,5-dihydroxybenzoic acid and the tyrosinase active site’s amino acid residues such as His85, His259, Phe264, Gly281, Ser282, and Val283 (Figure 2D and Table 4). On the other hand, the 2,3-dihydroxybenzoic acid docking pose only showed one possible hydrogen bond between the position 3 hydroxyl (-OH) group and His263, a catalytic residue of tyrosinase, and three additional hydrophobic interactions with Phe264, Gly281, and Val283, as shown in Figure 2D,E, respectively.

Interestingly, molecular docking suggested two possible binding conformations of genistein. The first conformation exhibited rings A and C of genistein docked into the active site (Figure 2G,H). In contrast, the second conformation only had ring B of genistein entering the catalytic pocket (Figure 2I,J). Additionally, the 3D and 2D molecular interaction analysis showed that these two docking possibilities exhibited significant differences in hydrogen bonds and hydrophobic interactions. For the first conformation, seven possible hydrogen bonds were proposed with rings A and C in the tyrosinase catalytic domain. Five hydrogen bonds were suggested from the 3D interaction analysis. Furthermore, in the 2D analysis, two copper ion interactions with two additional hydrophobic interactions were proposed, as shown in Figure 2G,H and Table 4. In contrast, the second conformation offered fewer hydrogen bonds (only two) with three additional hydrophobic interactions, as shown in Figure 2I,J and Table 4.

A further theoretically estimated free binding energy was calculated to assist the selection of a genistein docking pose more related to the authors’ anti-tyrosinase experiment and to support the observed molecular structure alteration between the hydroxybenzoic derivatives found earlier. The authors used two additional docking software programs (Autodock4 and Gnina) to obtain the theoretical binding energy. Notably, Gnina is a deep learning-integrated molecular docking software that can provide an additional convolutional neural network (CNN) score, indicating a probability of good docking pose on top of an improved conventional theoretically estimated binding score. Table 5 shows the estimated binding energies of all three metabolites, including 2,3-dihydroxybenzoic acid, 2,5-dihydroxybenzoic acid, and genistein (both docking conformations). The author used all data from the three metabolites to evaluate the correlation between the simulation and enzyme experiment. Based on the statistical correlation between the simulation and experimental data in Table 5, the second docked genistein conformation correlated more to an *in vitro* anti-tyrosinase assay in all obtained theoretically estimated free binding energy from three different software programs.

However, the Gnina CNN score suggested a higher score from the first docked genistein conformation, 0.55, than the second one, 0.45 (Table 5). This score indicates a higher probability that the first conformation will be a better pose according to a deep learning model. Therefore, the authors propose a mixing binding mode between genistein and tyrosinase with ring B inserted into the tyrosinase’s active site (the second conformation) as a predominant binding mode, while rings A and C are inserted (the first conformation) as a subsidiary binding mode. Finally, the authors predicted the optimum genistein conformation ratio using the results of the estimated binding energy from Gnina and Autodock4, since the two software programs provided a high correlation value of more than 0.9 between the theoretical simulation and biological experiment. Gnina’s estimated energy suggested a 95% second conformation with a 5% first conformation as the optimum ratio. On the other hand, Autodock4’s estimation suggested a 75% second conformation with a 25% first conformation as the optimum genistein conformation ratio. The prediction here is provided in the Supplementary File (Table S4 and Figures S17 and S18).

#### 3.4.2. Nonpolar Compound Analysis

**Figure 3:**
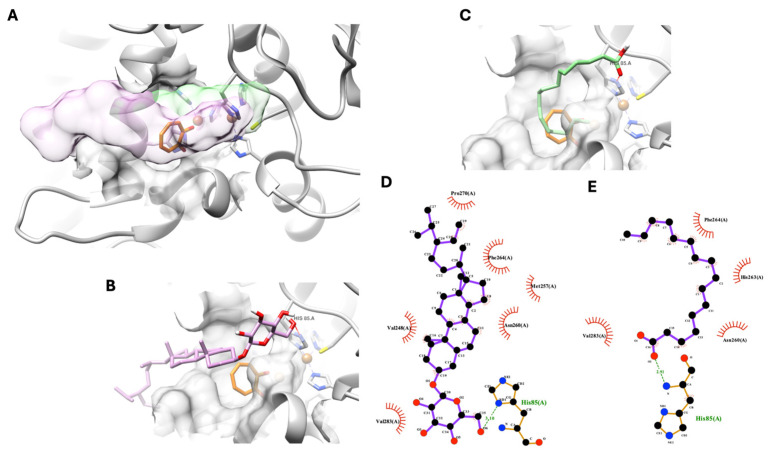
Selected docking poses of nonpolar compounds, *β*-sitosterol glucoside (pink) and palmitic acid (green), isolated from *D. heterocarpon* at the entrance of a tyrosinase’s catalytic pocket when tropolone (organ), a native ligand and known inhibitor, is located inside the pocket (**A**). The 3D and 2D interaction schemes between the tyrosinase’s catalytic pocket, *β*-sitosterol glucoside (**B**,**D**), and palmitic acid (**C**,**E**).

As shown in the previous section, two nonpolar compounds were isolated from *D. heterokaryon* and exhibited anti-tyrosinase activity. However, the theoretical docking simulation suggested that *β*-sitosterol glucoside and palmitic acid exhibited non-specific inhibitory behavior. As shown in Figure 3A below, both molecules’ chemical structures, *β*-sitosterol glucosidase in pink and palmitic acid in green, could not fit into the catalytic domain of tyrosinase. However, with one hydrogen bond at their polar head group with a tyrosinase’s amino acid, His85, from the outer side of the catalytic domain at the entrance of the tyrosinase’s active site (Figure 3B,C), both compounds could anchor their polar part of the molecule near the entrance area of the tyrosinase catalytic domain. Meanwhile, the rest of the molecule formed a hydrophobic interaction with non-catalytic residues of tyrosinase, completely blocking the active site of tyrosinase, as shown in Figure 3A,D,E. This theoretical interaction indicates a non-specific binding between the compound and protein.

## 4. Discussion

*D. heterocarpon* is a member of the Fabaceae family, which is known for its significant contributions to business, science, medicine, and culture. Several species of *Desmodium* have been used in traditional medicine to treat a variety of conditions, including convulsions, biliousness, cough, dysentery, diarrhea, bronchitis, typhoid, and asthma [33]. It is known that 56 species of *Desmodium* have biological activity and are used in traditional medicine. This genus has been used for more than 100 traditional therapeutic purposes from 43 different countries. There are 70 papers describing the genus’s traditional medicinal uses in India, 36 in China, 13 in Peru, 10 in Brazil, and 10 in Pakistan [8]. For *D. heterocarpon*, it exhibited strong antioxidant activity by a DPPH assay [33]. In addition, the leaf extract of *D. heterocarpon* showed the potential of antimicrobial activity against bacteria like *Bacillus cereus*, *S. aureus*, *Salmonella typhi*, *E. coli*, and *Klebsiella pneumonaie*, as well as fungi like *Aspergillus niger, Penicillium chrysogenum*, *Saccharomyces cerevisiae*, and *C. bicans* [34]. According to the Thai medical database, *D. heterocapon* roots can be used as an antihelminthic medicine by combining them with powdered *A. lacucha* Roxb and the root of *Aganosma marginata* (Roxb.) G.Don. The mixture is then boiled in water and consumed one tablespoon at a time, three times a day [35]. In accordance with the Plant Genetic Conservation Project, this plant is fed to sheep, goats, cattle, and buffalo by allowing the animals to graze on it [36]. However, there are limited reports of biological activity and none on the activity inhibitory effectiveness on the tyrosinase enzyme from *D. heterocarpon*. In this study, it was discovered that the three parts of roots and stem extracts effectively suppress tyrosinase enzyme activity. Following the separation of the significant compounds, six pure compounds were discovered such as genistein (compound **1**), hexadecanoic acid (compound **2**), salicylic acid (compound **3**), β-sitosterol-D-glucoside (compound **4**), 2,3-dihydroxybenzoic acid (compound **5**), and 2,5-dihydroxybenzoic acid (compound **6**).

2,5-dihydroxybenzoic acid, also known as gentisic acid, showed potential effects for tyrosinase inhibition with an IC_50_ of 57.38 µg/mL. In accordance with previous studies, the methyl ester of 2,5-dihydroxybenzoic acid functions as a pro-drug and releases hydroquinone, an inhibitor of tyrosinase. Additionally, compared to hydroxyquinone, methyl gentisate is less cytotoxic and mutagenic [37]. An *in vivo* study of melanin accumulation showed that it can be inhibited by gentisic acid. In the melanosomes of murine melanocyte cells, the methyl ester inhibited melanin synthesis by reducing tyrosinase synthesis or liveness without causing cytotoxicity [38]. While 2,3-dihydroxybenzoic acid’s structure was comparable to that of 2,5-dihydroxybenzoic acid, its ability to inhibit tyrosinase was not as strong (IC_50_ of 128.19 µg/mL). Therefore, the location of the hydroxyl group within the structure may have an impact on the enzyme’s activity. In this study, molecular docking has demonstrated the validity of this premise. Our results indicated that 2,5-dihydroxybenzoic acid showed more hydrogen bonds and hydrophobic bonds than 2,3-dihydroxybenzoic acid. Additionally, the structural molecular alignment between the selected docking poses of these two derivatives indicated a notable change in the carboxylic (-COOH) positions between two dihydroxybenzoic acids by 1.59 Å in the distance with an absolute 128-degree angle, leading to a non-favorable hydrogen bond between the carboxylic (-COOH) group from 2,3-dihydroxybenzoic acid and an Asn260 residue from tyrosinase, as shown in Figure 2B.

Genistein is also well known as an isoflavone. It is a phytoestrogen that offers heart system protection. Its glycoside can reverse UV-induced skin aging and stop the development of melanoma cells [39]. In an *in vitro* study, it showed weak anti-tyrosinase action (IC_50_ of 473.44 µM) in contrast to the positive control, kojic acids and aqueous extracts of *A. lacucha*. This result was consistent with the previous study’s finding that genistein had minimal anti-tyrosinase activity, with an IC_50_ of 362.54 µM [40] and 822 µM [41]. However, genistein can prevent animals or cells from producing melanin. It slowed the growth of melanoma cells that expressed EP3 by blocking the expression of IL-8 that PGE2 produced [42]. And it was discovered that 2% genistein might lower the amount of tyrosinase in guinea pigs’ skin pigmentation caused by ultraviolet B (UVB) radiation [43]. Those results suggested that the hydroxyl groups at positions C-6 and C-7 are crucial for anti-tyrosinase activity. Based on Masamoto et al. [44], this phenomenon seems to be a sufficient explanation. The anti-tyrosinase action was shown to be significantly influenced by the identical hydroxyl groups located at positions C-6 and C-7 of the coumarin skeleton. From the molecular docking studies, genistein provided two possible binding conformations. Rings A and C of genistein were seen to be docked onto the active site in the first conformation. In contrast, only genistein’s ring B entered the catalytic pocket in the second conformation. Furthermore, these two docking options demonstrated notable variations in hydrophobic and hydrogen bond interactions. Lastly, the higher binding energy values of the first conformation in Table 5 indicated that the first conformation was more suitable for modeling than the second conformation.

Hexadecanoic acid (compound **2**) or palmitic acid and β-sitosterol-D-glucoside (compound **4**) were nonpolar compounds isolated from *D. heterocarpon. In vitro*, they exhibited low inhibitory activity against tyrosinase. According to the outcomes of *in silico* tests, these compounds did not fit into the tyrosinase catalytic domain, indicating that the compounds and tyrosinase protein were bound non-specifically.

Salicylic acid (compound **3**) presented in such small amounts; therefore, its anti-tyrosinase activity was not tested. However, salicylic acid was earlier found to have an inhibitory concentration of 1.0 mM (138 µg/mL), which results in a 50% activity decrease. According to the analysis of its inhibition kinetics using Lineweaver–Burk plots, salicylic acid was a non-competitive inhibitor. Salicylic acid decreased the amount of oxygen consumed in the tyrosinase-catalyzed oxidation of L-DOPA [45]. Melanosome activity and melanogenesis in zebrafish and other melanocyte species are affected by salicylic acid found in ginseng roots. It demonstrated minimal cytotoxicity and decreased tyrosinase activity and melanin levels in B16F10 murine melanoma cells and normal human epidermal melanocytes, regardless of prior cell stimulation by the α-melanocyte-stimulating hormone [46].

The emergence of antibiotic-resistant microbes has restricted the availability of drugs for several infectious diseases. It is therefore crucial to investigate alternate antimicrobial drugs and treatments. Apart from their ability to inhibit enzymes, the pure compounds from *D. heterocarpon* also had the ability to prevent the growth of microbes. This study concentrated on cutaneous pathogens, which include fungus, yeasts, and bacteria. The first to infect a wound are often Gram-positive bacteria; subsequently, once the wound is healed, Gram-negative bacteria may be detected [47]. As secondary infections, molds and yeasts are especially dangerous for those on broad-spectrum antibiotic therapy. In this study, two phenolic acids, 2,3-dihydroxybenzoic acid (compound **5**) and 2,5-dihydroxybenzoic acid (compound **6**), were the active compounds that exhibited potential action with a low MIC and MBC/MFC. Previous reports have provided that 2,3-dihydroxybenzoic acid at a concentration of 315 ± 0.04 µg/mL inhibited the growth of *P. aeruginosa*, *Klebsiella pneumoniae*, *E. coli*, *Salmonella typhimurium*, and *S. aureus* [48]. At 30 mg/mL of 2,3-dihydroxybenzoic acid, which was isolated from the fruit extract of *Flacourtia inermis* Roxb, it showed antifungal action by preventing the growth of *A. fumigatus*, *A. flavus*, *A. niger*, and tested *Chrysosporium* species [31]. 2,5-dihydroxybenzoic acid (gentisic acid) exhibited antifungal activity. At a dosage of 5 mM, it inhibited *Rhizopus stolonifer* and *Botrytis* spore germination [49]. In Merkl et al.’s [50] research, 2,5-dihydroxybenzoic acid (MIC = 2.5 mM) reduced the growth of *B. aureus*, *L. monocytogenes*, and *E. coli*. 2,3-dihydroxybenzoic acid is a siderophore found naturally in plants and is also generated from the disodium salt of catechol. It has been given orally to thalassemia patients as an iron-chelating medication with less toxicity. Bacterial growth or biofilm development can be inhibited by 2,3-dihydroxybenzoic acid because this compound can interact with free iron in the environment, lowering the iron concentration to a point where it is not suitable for bacterial growth [51]. Additionally, the antibacterial action mechanism of phenolic acids may be connected to their undissociated phenolic acid forms. It penetrates the cell membrane by passive diffusion. This causes the cell membrane to be disrupted, which causes the outflow of vital intracellular components and ultimately leads to the acidification of the cytoplasm and death of microorganisms [52]. Moreover, in this study, 2,3-dihydroxybenzoic acid (compound **5**) and 2,5-dihydroxybenzoic acid (compound **6**) showed equal or similar MIC and MBC values, indicating that both compounds were active as bactericidal agents. The definition of bactericidal was indicated by the bactericidal antibiotic MBC being less than or equal to four folds above the MIC [53].

## 5. Conclusions

*D. heterocarpon* extracts tested in this study had the highest potent anti-tyrosinase activity. Among the tested extracts, the ethyl acetate extract and ethanol extract showed the highest efficacy in an *in vitro* experiment of anti-tyrosinase activity. These results could be related to the content of active compounds, particularly phenolic acids such as 2,3-dihydroxybenzoic acid (compound **5**) and 2,5-dihydroxybenzoic acid (compound **6**) (IC_50_ = 128.89 and 57.38 µg/mL). Molecular docking studies demonstrated that the position of the hydroxyl (-OH) in the structures of both compounds is an important mechanism in tyrosinase inhibitory activity. These pure compounds also have the advantage of being able to eradicate bacteria and fungus that cause disease on the skin. According to our present research, *D. heterocarpon* extract may be promising options for use in cosmetic applications or as a treatment for microbial infections.

## Figures and Tables

**Table 1:** The % yield and % tyrosinase inhibition of *D. heterocarpon* extracts.

Part of Extracts	Amount (g)	% Yield Base on Dry Weight of Plant	% Tyrosinase Inhibition at 20 µg/mL ± SD
petroleum ether extract	10.12	0.26	1.30 ± 3.36
ethyl acetate extract	20.43	0.54	74.71 ± 0.77
ethanol extract	188.73	4.96	60.41 ± 0.53
water extract	133.25	31.50	11.55 ± 2.94
kojic acid			87.37 ± 0.63
*Artocarpus lacucha*			96.98 ± 0.48

**Table 2:** IC_50_ values of pure compounds on tyrosinase inhibitory activity.

Compounds Name	IC_50_ (µg/mL)	IC_50_ (µM)
Genistein (compound 1)	127.83	473.44
Hexadecanoic acid (compound 2)	160.82	628.20
Salicylic acid (compound 3)	NT	NT
*β*-sitosterol-D-glucoside (compound 4)	221.99	385.39
2,3-dihydroxybenzoic acid (compound 5)	128.89	836.94
2,5-dihydroxybenzoic acid (compound 6)	57.38	372.59
Kojic acid ^P^	2.46	17.32
*Artocarpus lacucha* ^P,W^	0.15	-

^P^ = positive control; ^W^ = water extract; NT = not tested due to the trace amount of compound.

**Table 3:** MIC, MBC, and MFC values of pure compounds against pathogenic microorganisms.

Microorganism Strains	MIC/MBC or MFC (µg/mL) of Pure Compound						
	1	2	3	4	5	6	Antibiotic *
Gram-positive bacteria							
*S. aureus*	>100/>100	>100/>100	NT	>100/>100	15/15	5/5	0.25
MRSA	>100/>100	>100/>100	NT	>100/>100	15/15	15/35	0.5
*S. epidermidis*	>100/>100	>100/>100	NT	>100/>100	15/15	15/15	0.5
*P. acnes*	>100/>100	>100/>100	NT	>100/>100	15/20	30/40	0.5
Gram-negative bacteria							
*E. coli*	>100/>100	>100/>100	NT	>100/>100	15/15	20/35	0.5
*P. aeruginosa*	>100/>100	>100/>100	NT	>100/>100	15/20	20/35	0.5
Fungi							
*C. albicans*	>100/>100	>100/>100	NT	>100/>100	10/15	15/15	0.5
*M. gypseum*	>100/>100	>100/>100	NT	>100/>100	10/10	15/15	0.25
*T. rubrum*	>100/>100	>100/>100	NT	>100/>100	10/10	15/15	0.25
*T. mentagrophytes*	>100/>100	>100/>100	NT	>100/>100	10/10	15/15	0.25

* Antibiotics: oxacillin for *S. aureus*, *S. epidermidis*, *P. acnes*; vancomycin for MRSA; norfloxacin for *E. coli*, *P. aeruginosa*; amphotericin B for *C. albicans*; ketoconazole for *M. gypseum*, *T. rubrum*, *T. mentagrophytes*; NT = not tested due to the trace amount of compound.

**Table 4:** Summary of intermolecular interaction between selected polar compounds isolated from *D. heterocarpon* and tyrosinase.

Compounds	Selected Conformation	Position	Functional Group	Interaction Type	Interaction Number	Residues
2,5-dihydroxybenzoic acid	1	1	-COOH	H-bond	1	Asn260
		2	-OH	H-bond	1	His263
				Hydrophobic	6	His85, His259, Phe264, Gly281, Ser282, and Val283
2,3-dihydroxybenzoic acid	1	3	-OH	H-bond	1	His263
				Hydrophobic	3	Phe264, Gly281, and Val283
Genistein	2					
*Conformation 1*		5	-OH	H-bond	1	Met280
		7	-OH	H-bond	6	His61, His85 (2 bonds from 3D), His59, and 2 Cu ions (only from 2D analysis)
				Hydrophobic	2	Phe264 and Val283
*Conformation 2*		4′	-OH	H-bond	2	His263 and Cu ions (only from 2D analysis)
				Hydrophobic	3	His85, Phe264, and Val283

**Table 5:** Overview of the correlation between an *in vitro* experiment and the estimated binding energy of three isolated polar compounds at the tyrosinase active site obtained from different docking software programs, including a convolutional neural network from Gnina for docking pose evaluation.

Name	μM *	Vina (Kcal/Mol)	AD4 (Kcal/Mol)	Gnina	
				Binding Energy (Kcal/Mol)	CNN Score
2,5-dihydroxybenzoic acid	372.59				
Docking		−5.95	−6.18	−6.53	0.65
2,3-dihydroxybenzoic acid	836.94				
Docking		−5.43	−5.70	−6.02	0.63
Genistein	473.44				
Docking 1		−6.86	−6.66	−7.96	0.55
Experiment: docking1 correlation		0.63	0.74	0.54	
Docking 2		−5.48	−5.90	−6.34	0.48
Experiment: docking2 correlation		0.73	0.90 **	0.99^**^	

* = *in vitro* tyrosinase binding assay, while ** = correlation value equal or higher than 0.90. Vina = Autodock Vina; AD4 = Autodock4; and CNN = convolutional neural network.

## Data Availability

Data are contained within the article and Supplementary Materials.

## Supplementary Materials

The following supporting information can be downloaded at: https://www.mdpi.com/article/10.3390/life14111400/s1, Table S1: List of 114 Fabaceae plant samples; Table S2: Chemical IDs of used compounds in this study; Table S3: List of Fabaceae plants and their preliminary screening of tyrosinase inhibitory activity; Table S4: Calculated genistein conformation ratio optimization using a linear regression function under biological and virtual correlation; Figure S1: The solvent extraction of *Desmodium heterocarpon*; Figure S2: Separation processes of compounds 1 to 6 from crude extract; Figure S3: ^1^H-NMR spectrum of genistein (compound **1**) (CD_3_OD; 500 MHz); Figure S4: ^13^C-NMR spectrum of genistein (compound **1**) (CD_3_OD; 125 MHz); Figure S5: ^1^H-NMR spectrum of hexadecanoic acid (compound **2**) (CDCl_3_; 500 MHz); Figure S6: ^1^H-NMR spectrum of salicylic acid (compound **3**) (CDCl_3_; 500 MHz); Figure S7: ^13^C-NMR spectrum of salicylic acid (compound **3**) (CDCl_3_; 125 MHz); Figure S8: ^1^H-^1^H NMR spectrum of salicylic acid (compound **3**) (CDCl_3_); Figure S9: ^1^H-NMR spectrum of β-sitosterol-D-glucoside (compound **4**) (DMSO; 500 MHz); Figure S10: ^13^C-NMR spectrum of β-sitosterol-D-glucoside (compound **4**) (DMSO; 125 MHz); Figure S11: ^1^H-NMR spectrum of 2,3-dihydroxybenzoic acid (compound **5**) (DMSO; 500 MHz); Figure S12: ^13^C-NMR spectrum of 2,3-dihydroxybenzoic acid (compound **5**) (DMSO; 125 MHz); Figure S13: ^1^H-^1^H NMR spectrum of 2,3-dihydroxybenzoic acid (compound **5**) (DMSO); Figure S14: ^1^H-NMR spectrum of 2,5-dihydroxybenzoic acid (compound **6**) (DMSO; 500 MHz); Figure S15: ^13^C-NMR spectrum of 2,5-dihydroxybenzoic acid (compound **6**) (DMSO; 125 MHz); Figure S16: Docking validation via re-docking the extracted native ligand back (black) into its original position (organ). PDB ID: 2y9x is used as a target enzyme, fungal tyrosinase. The RMSD between the re-docked native ligand and its original pose is 1.070 Å; Figure S17: Predictive genistein conformation optimization guided by linear correlation between experimental data (IC_50_ value) and estimated binding energy from Autodock Vina; Figure S18: Predictive genistein conformation optimization guided by linear correlation between experimental data (IC_50_ value) and estimated binding energy from Gnina.
