# Supplementary material for: Phytochemical Investigation and Biological Activities of Desmodium heterocarpon Extract as Anti-Tyrosinase: Isolation of Natural Compounds, In Vitro and In Silico Study
Source: Life (Basel). 2024 Oct 31;14(11):1400. doi: 10.3390/life14111400 (PMC11595612; doi:10.3390/life14111400)
Supplement: Supplementary file 1 [file life-14-01400-s001.zip › life-3230933-supplementary.pdf]

Supplementary Materials

Table S1 List of 114 Fabaceae plant samples

No.	Code	Thai name	Part used	Scientific name	Source
1	BA-001-B		branch	*Bauhinia acuminata*	Southern Thai Literary Botanical Garden, Songkhla
2	BA-002-L		leaf		
3	BP-003-B		branch	*Bauhinia purpurea*	Prince of Songkla University, Songkhla
4	BP-004-L		leaf		
5	BP-005-F		fruit		
6	CC-006-B		branch	*Caesalpinia coriaria*	Southern Thai Literary Botanical Garden, Songkhla
7	CC-007-L		leaf		
8	CC-008-F		fruit		
9	BM-009-B		branch	*Butea monosperma*	Southern Thai Literary Botanical Garden, Songkhla
10	BM-010-L		leaf		
11	PP-011-B		branch	*Peltophorum pterocarpum*	Southern Thai Literary Botanical Garden, Songkhla
12	PP-012-L		leaf		
13	TI-013-B		branch	*Tamarindus indica*	Prince of Songkla University, Songkhla
14	TI-014-L		leaf		
15	CB-015-B		branch	*Caesalpinia bonduc*	Southern Thai Literary Botanical Garden, Songkhla
16	CB-016-L		leaf		
17	SG-017-B		branch	*Senna garrettiana*	Southern Thai Literary Botanical Garden, Songkhla
18	SG-018-L		leaf		
19	SI-019-B		branch	*Saraca indica*	Prince of Songkla University, Songkhla
20	SI-020-L		leaf		
21	LL-021-B		branch	*Leucaena leucocephala*	Muang, Songkhla
22	LL-022-L		leaf		
23	LL-023-F		fruit		
24	SS-024-B		branch	*Senna siamea*	Prince of Songkla University, Songkhla
25	SS-025-L		leaf		
26	CF-026-L		leaf	*Cassia fistula*	Prince of Songkla University, Songkhla
27	CF-027-F		fruit		
28	SGr-028-B		branch	*Sesbania grandiflora*	Muang, Songkhla
29	SGr-029-L		leaf		
30	SSa-030-B		branch	*Samanea saman*	Muang, Songkhla
31	SSa-031-L		leaf		
32	CS-032-W		wood	*Caesalpinia sappan*	Southern Thai Literary Botanical Garden, Songkhla
33	CS-033-L		leaf		
34	AP-034-B		branch	*Adenanthera pavonina*	Prince of Songkla University, Songkhla
35	AP-035-L		leaf		

36	PD-036-B		branch	*Pithecellobium dulce*	Prince of Songkla University, Songkhla
37	PD-037-L		leaf		
38	DR-038-B		branch	*Delonix regia*	Prince of Songkla University, Songkhla
39	DR-039-L		leaf		
40	DR-040-FL		flower		
41	BS-041-S		stem	*Bauhinia scandens*	Southern Thai Literary Botanical Garden, Songkhla
42	BS-042-L		leaf		
43	BT-043-B		branch	*Bauhinia tomentosa*	Southern Thai Literary Botanical Garden, Songkhla
44	BT-044-L		leaf		
45	BP-045-B		branch	*Bauhinia pottsii*	Hala-bala forest, Narathiwat
46	BP-046-L		leaf		
47	BSp-047-B		branch	*Bauhinia* spp.	Southern Thai Literary Botanical Garden, Songkhla
48	BSp-048-L		leaf		
49	BI-049-B		branch	*Bauhinia integrifolia*	Hala-bala forest, Narathiwat
50	BI-050-L		leaf		
51	BAu-051-B		branch	*Bauhinia aureifolia*	Southern Thai Literary Botanical Garden, Songkhla / Hala-bala forest, Narathiwat
52	BAu-052-L		leaf		
53	BF-053-B		branch	*Bauhinia ferruginea*	Hala-bala forest, Narathiwat
54	BF-054-L		leaf		
55	BG-055-B		branch	*Bauhinia glauca*	Hala-bala forest, Narathiwat
56	BG-056-L		leaf		
57	BSp-057-B		branch	*Bauhinia involucellata*	Southern Thai Literary Botanical Garden, Songkhla
58	BSp-058-L		leaf		
59	BSp-059-S		stem	*Bauhinia* spp.	Southern Thai Literary Botanical Garden, Songkhla
60	BSp-060-L		leaf		
61	AF-061-F		fruit	*Acacia farnesiana*	Southern Thai Literary Botanical Garden, Songkhla
62	DH-062-RS		root and stem	*Desmodium heterocarpon*	Southern Thai Literary Botanical Garden, Songkhla
63	DH-063-L		leaf		
64	AM-064-B		branch	*Afgekia mahidolae*	Southern Thai Literary Botanical Garden, Songkhla
65	AM-065-L		leaf		
66	AM-066-FL		flower		
67	DS-067-B		branch	*Derris scandens*	Southern Thai Literary Botanical Garden, Songkhla
68	DS-068-L		leaf		
69	APr-069-B		branch	*Albizia procera*	Southern Thai Literary Botanical Garden, Songkhla
70	APr-070-L		leaf		
71	DC-071-B		branch	*Dalbergia cochinchinensis*	Southern Thai Literary Botanical Garden, Songkhla
72	DC-072-L		leaf		
73	BV-073-B		branch	*Bauhinia variegata*	Central Thai Literary Botanical Garden, Ratchaburi
74	BV-074-L		leaf		
75	BB-075-B		branch	*Bauhinia bracteata*	Central Thai Literary Botanical Garden, Ratchaburi
76	BB-076-L		leaf		
77	BW-077-B		branch	*Bauhinia winitii*	Central Thai Literary Botanical Garden, Ratchaburi

78	BW-078-L		leaf		
79	CT-079-L		leaf	*Clitoria ternatea*	Muang, Surat Thani
80	CT-080-FL		flower		
81	AO-081-B		branch	*Albizia odoratissima*	Southern Thai Literary Botanical Garden, Songkhla
82	AO-082-L		leaf		
83	DCa-083-B		branch	*Dalbergia candenatensis*	Southern Thai Literary Botanical Garden, Songkhla
84	DCa-084-L		leaf		
85	DP-085-B		branch	*Dalbergia parviflora*	Southern Thai Literary Botanical Garden, Songkhla
86	DP-086-L		leaf		
87	DN-087-B		branch	*Dalbergia nigrescens*	Southern Thai Literary Botanical Garden, Songkhla
88	DN-088-L		leaf		
89	AMy-089-B		branch	*Albizia myriophylla*	Southern Thai Literary Botanical Garden, Songkhla
90	AMy-090-L		leaf		
91	MA-091-B		branch	*Millettia atropurpurea*	Southern Thai Literary Botanical Garden, Songkhla
92	MA-092-L		leaf		
93	PS-093-B		branch	*Phyllocarpus septentrionalis*	Southern Thai Literary Botanical Garden, Songkhla
94	PS-094-L		leaf		
95	CH-095-B		branch	*Calliandra haematocephala*	Southern Thai Literary Botanical Garden, Songkhla
96	CH-096-L		leaf		
97	AMi-097-B		branch	*Adenanthera microsperma*	Southern Thai Literary Botanical Garden, Songkhla
98	Ami-098-L		leaf		
99	PDa-099-B		branch	*Peltophorum dasyrachis*	Southern Thai Literary Botanical Garden, Songkhla
100	PDa-100-L		leaf		
101	APu-101-S		stem	*Abrus pulchellus*	Southern Thai Literary Botanical Garden, Songkhla
102	Apu-102-L		leaf		
103	KE-103-B		branch	*Koompassia excelsa*	Southern Thai Literary Botanical Garden, Songkhla
104	KE-104-L		leaf		
105	ST-105-B		branch	*Saraca thaipingensis*	Southern Thai Literary Botanical Garden, Songkhla
106	ST-106-L		leaf		
107	ST-107-FL		flower		
108	ST-108-F		fruit		
109	AC-109-B		branch	*Acacia concinna*	Southern Thai Literary Botanical Garden, Songkhla
110	AC-110-L		leaf		
111	BMa-111-L		leaf	*Bauhinia malabarica*	Southern Thai Literary Botanical Garden, Songkhla
112	ML-112-B		branch	*Millettia leucantha*	Southern Thai Literary Botanical Garden, Songkhla
113	ML-113-L		leaf		
114	ML-114-F		fruit		

Table S2. Chemical IDs of used compounds in this study.

**No**	**Name**	**PubChem ID**
1	Kojic acid	3840
2	2,3-Dihydroxybenzoic acid	19
3	2,5-Dihydroxybenzoic acid	3469
4	Genistein	5280961
5	β-Sitosterol-D-glucoside	481107568
6	Palmetic acid	985

Table S3 List of Fabaceae plants and their preliminary screening of tyrosinase inhibitory activity.

**No.**	**Code**	**Part use**	**Scientific name**	**% Tyrosinase inhibition at 20 μg/mL ± SD**
1	BA-001-B	branch	*Bauhinia acuminata*	7.96 ± 3.55
2	BA-002-L	leaf		10.41 ± 1.62
3	BP-003-B	branch	*Bauhinia purpurea*	4.91 ± 3.57
4	BP-004-L	leaf		33.70 ± 1.27
5	BP-005-F	fruit		4.52 ± 3.55
6	CC-006-B	branch	*Caesalpinia coriaria*	13.97 ± 1.88
7	CC-007-L	leaf		15.39 ± 0.09
8	CC-008-F	fruit		1.25 ± 0.95
9	BM-009-B	branch	*Butea monosperma*	0.53 ± 2.88
10	BM-010-L	leaf		19.33 ± 3.94
11	PP-011-B	branch	*Peltophorum pterocarpum*	9.00 ± 3.64
12	PP-012-L	leaf		2.32 ± 1.19
13	TI-013-B	branch	*Tamarindus indica*	1.68 ± 1.48
14	TI-014-L	leaf		34.32 ± 3.58
15	CB-015-B	branch	*Caesalpinia bonduc*	15.23 ± 3.44
16	CB-016-L	leaf		8.75 ± 1.53
17	SG-017-B	branch	*Senna garrettiana*	9.91 ± 2.72
18	SG-018-L	leaf		5.57 ± 1.96
19	SI-019-B	branch	*Saraca indica*	7.32 ± 3.53
20	SI-020-L	leaf		18.44 ± 1.89
21	LL-021-B	branch	*Leucaena leucocephala*	27.73 ± 1.27
22	LL-022-L	leaf		-11.34 ± 1.04
23	LL-023-F	fruit		-9.83 ± 1.95
24	SS-024-B	branch	*Senna siamea*	18.67 ± 2.45
25	SS-025-L	leaf		10.11 ± 3.06
26	CF-026-L	leaf	*Cassia fistula*	7.97 ± 0.68
27	CF-027-Fr	fruit		-6.36 ± 3.10
28	SGr-028-B	branch	*Sesbania grandiflora*	-7.02 ± 1.43
29	SGr-029-L	leaf		1.88 ± 0.43
30	SSa-030-B	branch	*Samanea saman*	-3.01 ± 1.63

31	SSa-031-L	leaf		4.36 ± 3.07
32	CS-032-W	wood	*Caesalpinia sappan*	0.16 ± 1.77
33	CS-033-L	leaf		7.50 ± 2.62
34	AP-034-B	branch	*Adenanthera pavonina*	4.19 ± 1.15
35	AP-035-L	leaf		11.73 ± 1.40
36	PD-036-B	branch	*Pithecellobium dulce*	5.59 ± 3.54
37	PD-037-L	leaf		23.28 ± 2.22
38	DR-038-B	branch	*Delonix regia*	8.14 ± 1.09
39	DR-039-L	leaf		2.58 ± 1.46
40	DR-040-F	flower		-8.46 ± 3.08
41	BS-041-S	stem	*Bauhinia scandens*	3.84 ± 3.04
42	BS-042-L	leaf		11.59 ± 1.85
43	BT-043-B	branch	*Bauhinia tomentosa*	-3.80 ± 2.56
44	BT-044-L	leaf		9.81 ± 3.13
45	BP-045-B	branch	*Bauhinia pottsii*	-10.85 ± 3.48
46	BP-046-L	leaf		-14.61 ± 1.49
47	BSp-047-B	branch	*Bauhinia* spp.	-6.30 ± 3.09
48	BSp-048-L	leaf		-4.78 ± 3.53
49	BI-049-B	branch	*Bauhinia integrifolia*	8.71 ± 3.98
50	BI-050-L	leaf		2.41 ± 3.55
51	BAu-051-B	branch	*Bauhinia aureifolia*	27.41 ± 2.37
52	BAu-052-L	leaf		7.26 ± 3.17
53	BF-053-B	branch	*Bauhinia ferruginea*	3.53 ± 1.25
54	BF-054-L	leaf		-10.43 ± 2.57
55	BG-055-B	branch	*Bauhinia glauca*	-10.61 ± 3.09
56	BG-056-L	leaf		-0.75 ± 3.50
57	BIn-057-B	branch	*Bauhinia involucellata*	-2.98 ± 1.97
58	BIn-058-L	leaf		-3.04 ± 2.78
59	BSp-059-S	stem	*Bauhinia* spp.	14.02 ± 3.40
60	BSp-060-L	leaf		19.24 ±1.92
61	AF-061-F	fruit	*Acacia farnesiana*	8.45 ± 2.14
**62**	**DH-062-RS**	**root, stem**	***Desmodium heterocarpon***	**90.96 ± 1.85**
63	DH-063-L	leaf		30.08 ± 1.67
64	AM-064-B	branch	*Afgekia mahidolae*	29.12 ± 2.38
65	AM-065-L	leaf		-123.31 ± 3.81
66	AM-066-FL	flower		4.17 ± 3.64
67	DS-067-B	branch	*Derris scandens*	14.81 ± 3.22
68	DS-068-L	leaf		6.93 ± 1.74
69	APr-069-B	branch	*Albizia procera*	-141.69 ± 3.95
70	APr-070-L	leaf		30.90 ± 4.65
71	DC-071-B	branch	*Dalbergia cochinchinensis*	8.50 ± 1.96
72	DC-072-L	leaf		43.76 ± 2.46
73	BV-073-B	branch	*Bauhinia variegata*	27.61 ± 2.75
74	BV-074-L	leaf		1.94 ± 4.08
75	BB-075-B	branch	*Bauhinia bracteata*	11.68 ± 3.49
76	BB-076-L	leaf		7.44 ± 3.12
77	BW-077-B	branch	*Bauhinia winitii*	19.27 ± 2.43
78	BW-078-L	leaf		15.42 ±3.64
78	CT-079-L	leaf	*Clitoria ternatea*	23.72 ± 1.87
80	CT-080-FL	flower		3.51 ± 3.77
81	AO-081-B	branch	*Albizia odoratissima*	-136.35 ± 2.76
82	AO-082-L	leaf		-171.16 ± 4.39

83	DCa-083-B	branch	*Dalbergia candenatensis*	3.41 ± 2.45
84	DCa-084-L	leaf		4.99 ± 2.23
85	DP-085-B	branch	*Dalbergia parvifiora*	8.25 ± 1.93
86	DP-086-L	leaf		46.81 ± 3.31
87	DN-087-B	branch	*Dalbergia nigrescens*	6.52 ± 1.38
88	DN-088-L	leaf		-0.67 ± 3.93
89	AMy-089-B	branch	*Albizia myriophylla*	4.64 ± 1.23
90	AMy-090-L	leaf		-3.75 ± 2.11
91	MA-091-B	branch	*Millettia atropurpurea*	-2.18 ± 3.14
92	MA-092-L	leaf		-2.09 ± 4.86
93	PS-093-B	branch	*Phyllocarpus septentrionalis*	-3.92 ± 3.21
94	PS-094-L	leaf		3.62 ± 3.81
95	CH-095-B	branch	*Calliandra haematocephala*	12.06 ± 4.22
96	CH-096-L	leaf		6.31 ± 3.26
97	AMi-097-B	branch	*Adenanthera microsperma*	3.16 ±1.91
98	AMi-098-L	leaf		-1.12 ± 3.47
99	PDa-099-B	branch	*Peltophorum dasyrachis*	4.13 ± 3.82
100	PDa-100-L	leaf		10.37 ± 2.57
101	APu-101-S	stem	*Abrus pulchellus*	17.67 ± 4.14
102	APu-102-L	leaf		21.26 ± 3.45
103	KE-103-B	branch	*Koompassia excelsa*	-3.30 ± 4.57
104	KE-104-L	leaf		-6.45 ± 3.62
105	ST-105-B	branch	*Saraca thaipingensis*	-6.29 ± 4.09
106	ST-106-L	leaf		-4.78 ± 4.52
107	ST-107-FL	flower		-2.98 ± 1.97
108	ST-108-F	fruit		-8.50 ± 1.79
109	AC-109-B	branch	*Acacia concinna*	21.74 ± 3.11
110	AC-110-L	leaf		14.28 ± 3.94
111	BMa-111-L	leaf	*Bauhinia malabarica*	-5.24 ± 3.74
112	ML-112-B	branch	*Millettia leucantha*	4.32 ± 3.38
113	ML-113-L	leaf		1.22 ± 1.92
114	ML-114-F	fruit		-0.45 ± 1.03
	Kojic acid[P]			92.29 ± 1.93
	Artocarpus lacucha[P]			98.82 ± 0.83

Table S4. Calculated genistein conformation ratio optimization using a linear regression function under biological and virtual correlation.

No	Ratio		Docking software					
	Conformation 2	Conformation 1	AD4			Gnina		
1	1,00	0,00	-5,90	0,00	-5,90	-6,34	0,00	-6,34
2	0,99	0,01	-5,84	-0,07	-5,91	-6,28	-0,08	-6,36
3	0,98	0,02	-5,78	-0,13	-5,92	-6,21	-0,16	-6,37
4	0,97	0,03	-5,72	-0,20	-5,92	-6,15	-0,24	-6,39
5	0,96	0,04	-5,66	-0,27	-5,93	-6,09	-0,32	-6,40
6	0,95	0,05	-5,61	-0,33	-5,94	-6,02	-0,40	-6,42
7	0,94	0,06	-5,55	-0,40	-5,95	-5,96	-0,48	-6,44
8	0,93	0,07	-5,49	-0,47	-5,95	-5,90	-0,56	-6,45
9	0,92	0,08	-5,43	-0,53	-5,96	-5,83	-0,64	-6,47
10	0,91	0,09	-5,37	-0,60	-5,97	-5,77	-0,72	-6,49
11	0,90	0,10	-5,31	-0,67	-5,98	-5,71	-0,80	-6,50
12	0,89	0,11	-5,25	-0,73	-5,98	-5,64	-0,88	-6,52
13	0,88	0,12	-5,19	-0,80	-5,99	-5,58	-0,96	-6,53
14	0,87	0,13	-5,13	-0,87	-6,00	-5,52	-1,03	-6,55
15	0,86	0,14	-5,07	-0,93	-6,01	-5,45	-1,11	-6,57
16	0,85	0,15	-5,02	-1,00	-6,01	-5,39	-1,19	-6,58
17	0,84	0,16	-4,96	-1,07	-6,02	-5,33	-1,27	-6,60
18	0,83	0,17	-4,90	-1,13	-6,03	-5,26	-1,35	-6,62
19	0,82	0,18	-4,84	-1,20	-6,04	-5,20	-1,43	-6,63
20	0,81	0,19	-4,78	-1,27	-6,04	-5,14	-1,51	-6,65
21	0,80	0,20	-4,72	-1,33	-6,05	-5,07	-1,59	-6,66
22	0,79	0,21	-4,66	-1,40	-6,06	-5,01	-1,67	-6,68
23	0,78	0,22	-4,60	-1,47	-6,07	-4,95	-1,75	-6,70
24	0,77	0,23	-4,54	-1,53	-6,07	-4,88	-1,83	-6,71
25	0,76	0,24	-4,48	-1,60	-6,08	-4,82	-1,91	-6,73
26	0,75	0,25	-4,43	-1,67	-6,09	-4,76	-1,99	-6,75
27	0,74	0,26	-4,37	-1,73	-6,10	-4,69	-2,07	-6,76

28	0,73	0,27	-4,31	-1,80	-6,11	-4,63	-2,15	-6,78
29	0,72	0,28	-4,25	-1,86	-6,11	-4,56	-2,23	-6,79
30	0,71	0,29	-4,19	-1,93	-6,12	-4,50	-2,31	-6,81
31	0,70	0,30	-4,13	-2,00	-6,13	-4,44	-2,39	-6,83
32	0,69	0,31	-4,07	-2,06	-6,14	-4,37	-2,47	-6,84
33	0,68	0,32	-4,01	-2,13	-6,14	-4,31	-2,55	-6,86
34	0,67	0,33	-3,95	-2,20	-6,15	-4,25	-2,63	-6,87
35	0,66	0,34	-3,89	-2,26	-6,16	-4,18	-2,71	-6,89
36	0,65	0,35	-3,84	-2,33	-6,17	-4,12	-2,79	-6,91
37	0,64	0,36	-3,78	-2,40	-6,17	-4,06	-2,87	-6,92
38	0,63	0,37	-3,72	-2,46	-6,18	-3,99	-2,95	-6,94
39	0,62	0,38	-3,66	-2,53	-6,19	-3,93	-3,02	-6,96
40	0,61	0,39	-3,60	-2,60	-6,20	-3,87	-3,10	-6,97
41	0,60	0,40	-3,54	-2,66	-6,20	-3,80	-3,18	-6,99
42	0,59	0,41	-3,48	-2,73	-6,21	-3,74	-3,26	-7,00
43	0,58	0,42	-3,42	-2,80	-6,22	-3,68	-3,34	-7,02
44	0,57	0,43	-3,36	-2,86	-6,23	-3,61	-3,42	-7,04
45	0,56	0,44	-3,30	-2,93	-6,23	-3,55	-3,50	-7,05
46	0,55	0,45	-3,25	-3,00	-6,24	-3,49	-3,58	-7,07
47	0,54	0,46	-3,19	-3,06	-6,25	-3,42	-3,66	-7,09
48	0,53	0,47	-3,13	-3,13	-6,26	-3,36	-3,74	-7,10
49	0,52	0,48	-3,07	-3,20	-6,26	-3,30	-3,82	-7,12
50	0,51	0,49	-3,01	-3,26	-6,27	-3,23	-3,90	-7,13
51	0,50	0,50	-2,95	-3,33	-6,28	-3,17	-3,98	-7,15
52	0,49	0,51	-2,89	-3,40	-6,29	-3,11	-4,06	-7,17
53	0,48	0,52	-2,83	-3,46	-6,30	-3,04	-4,14	-7,18
54	0,47	0,53	-2,77	-3,53	-6,30	-2,98	-4,22	-7,20
55	0,46	0,54	-2,71	-3,60	-6,31	-2,92	-4,30	-7,21
56	0,45	0,55	-2,66	-3,66	-6,32	-2,85	-4,38	-7,23
57	0,44	0,56	-2,60	-3,73	-6,33	-2,79	-4,46	-7,25
58	0,43	0,57	-2,54	-3,80	-6,33	-2,73	-4,54	-7,26

59	0,42	0,58	-2,48	-3,86	-6,34	-2,66	-4,62	-7,28
60	0,41	0,59	-2,42	-3,93	-6,35	-2,60	-4,70	-7,30
61	0,40	0,60	-2,36	-4,00	-6,36	-2,54	-4,78	-7,31
62	0,39	0,61	-2,30	-4,06	-6,36	-2,47	-4,86	-7,33
63	0,38	0,62	-2,24	-4,13	-6,37	-2,41	-4,94	-7,34
64	0,37	0,63	-2,18	-4,20	-6,38	-2,35	-5,01	-7,36
65	0,36	0,64	-2,12	-4,26	-6,39	-2,28	-5,09	-7,38
66	0,35	0,65	-2,07	-4,33	-6,39	-2,22	-5,17	-7,39
67	0,34	0,66	-2,01	-4,40	-6,40	-2,16	-5,25	-7,41
68	0,33	0,67	-1,95	-4,46	-6,41	-2,09	-5,33	-7,43
69	0,32	0,68	-1,89	-4,53	-6,42	-2,03	-5,41	-7,44
70	0,31	0,69	-1,83	-4,60	-6,42	-1,97	-5,49	-7,46
71	0,30	0,70	-1,77	-4,66	-6,43	-1,90	-5,57	-7,47
72	0,29	0,71	-1,71	-4,73	-6,44	-1,84	-5,65	-7,49
73	0,28	0,72	-1,65	-4,80	-6,45	-1,78	-5,73	-7,51
74	0,27	0,73	-1,59	-4,86	-6,45	-1,71	-5,81	-7,52
75	0,26	0,74	-1,53	-4,93	-6,46	-1,65	-5,89	-7,54
76	0,25	0,75	-1,48	-4,99	-6,47	-1,59	-5,97	-7,55
77	0,24	0,76	-1,42	-5,06	-6,48	-1,52	-6,05	-7,57
78	0,23	0,77	-1,36	-5,13	-6,49	-1,46	-6,13	-7,59
79	0,22	0,78	-1,30	-5,19	-6,49	-1,39	-6,21	-7,60
80	0,21	0,79	-1,24	-5,26	-6,50	-1,33	-6,29	-7,62
81	0,20	0,80	-1,18	-5,33	-6,51	-1,27	-6,37	-7,64
82	0,19	0,81	-1,12	-5,39	-6,52	-1,20	-6,45	-7,65
83	0,18	0,82	-1,06	-5,46	-6,52	-1,14	-6,53	-7,67
84	0,17	0,83	-1,00	-5,53	-6,53	-1,08	-6,61	-7,68
85	0,16	0,84	-0,94	-5,59	-6,54	-1,01	-6,69	-7,70
86	0,15	0,85	-0,89	-5,66	-6,55	-0,95	-6,77	-7,72
87	0,14	0,86	-0,83	-5,73	-6,55	-0,89	-6,85	-7,73
88	0,13	0,87	-0,77	-5,79	-6,56	-0,82	-6,93	-7,75
89	0,12	0,88	-0,71	-5,86	-6,57	-0,76	-7,00	-7,77

90	0,11	0,89	-0,65	-5,93	-6,58	-0,70	-7,08	-7,78
91	0,10	0,90	-0,59	-5,99	-6,58	-0,63	-7,16	-7,80
92	0,09	0,91	-0,53	-6,06	-6,59	-0,57	-7,24	-7,81
93	0,08	0,92	-0,47	-6,13	-6,60	-0,51	-7,32	-7,83
94	0,07	0,93	-0,41	-6,19	-6,61	-0,44	-7,40	-7,85
95	0,06	0,94	-0,35	-6,26	-6,61	-0,38	-7,48	-7,86
96	0,05	0,95	-0,30	-6,33	-6,62	-0,32	-7,56	-7,88
97	0,04	0,96	-0,24	-6,39	-6,63	-0,25	-7,64	-7,90
98	0,03	0,97	-0,18	-6,46	-6,64	-0,19	-7,72	-7,91
99	0,02	0,98	-0,12	-6,53	-6,64	-0,13	-7,80	-7,93
100	0,01	0,99	-0,06	-6,59	-6,65	-0,06	-7,88	-7,94
101	0,00	1,00	0,00	-6,66	-6,66	0,00	-7,96	-7,96

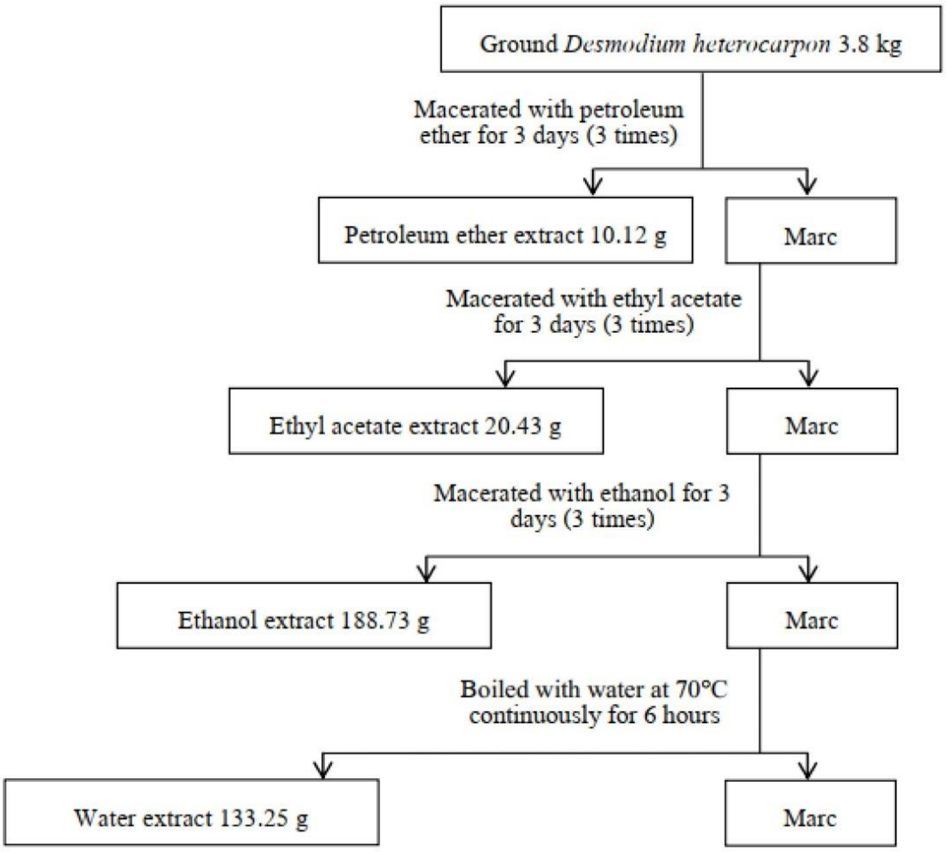

Figure S1. The solvent extraction of *Desmodium heterocarpon*
(Marc = Residue)

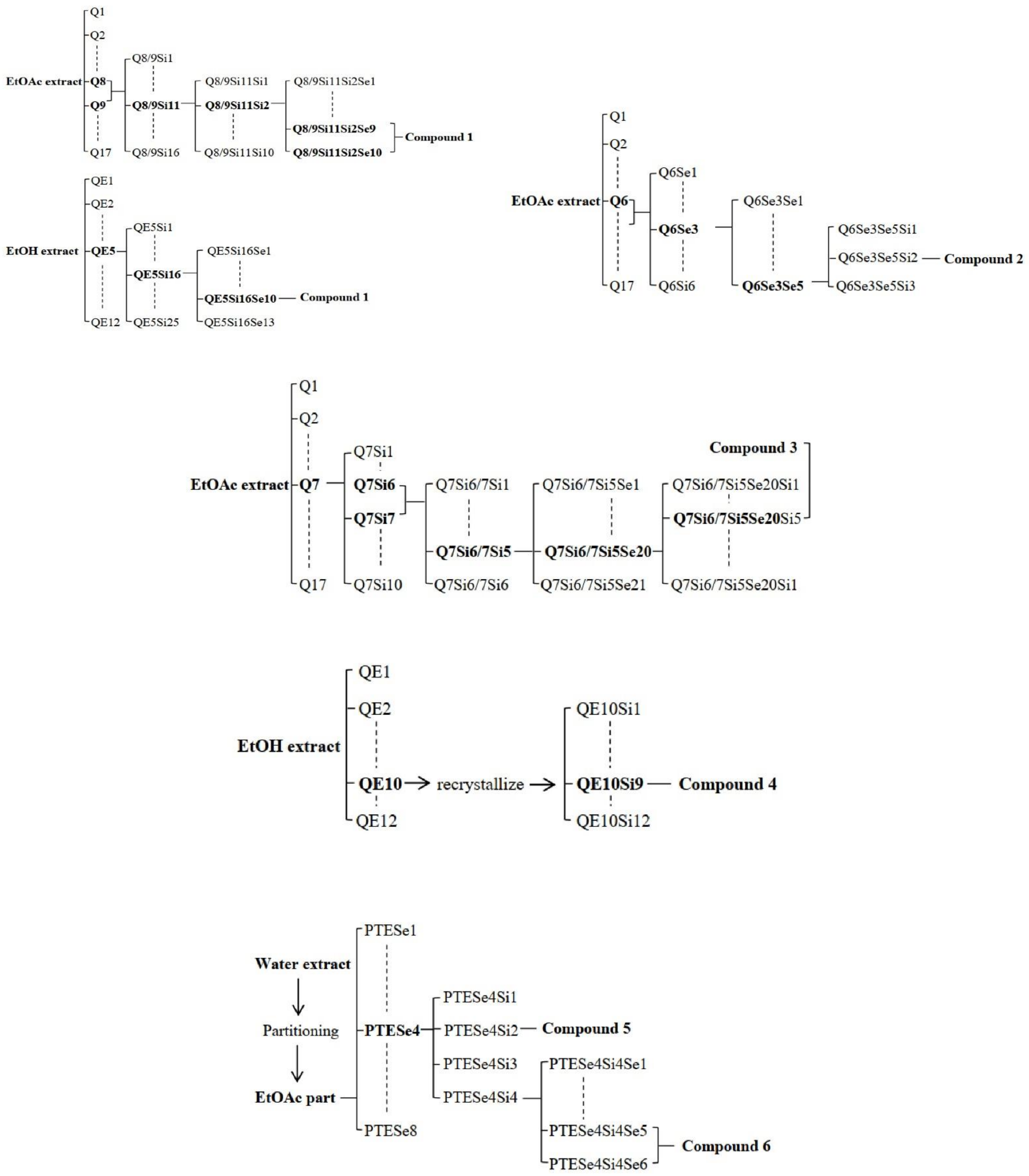

Figure S2. Separation processes of compounds 1 to 6 from crude extract

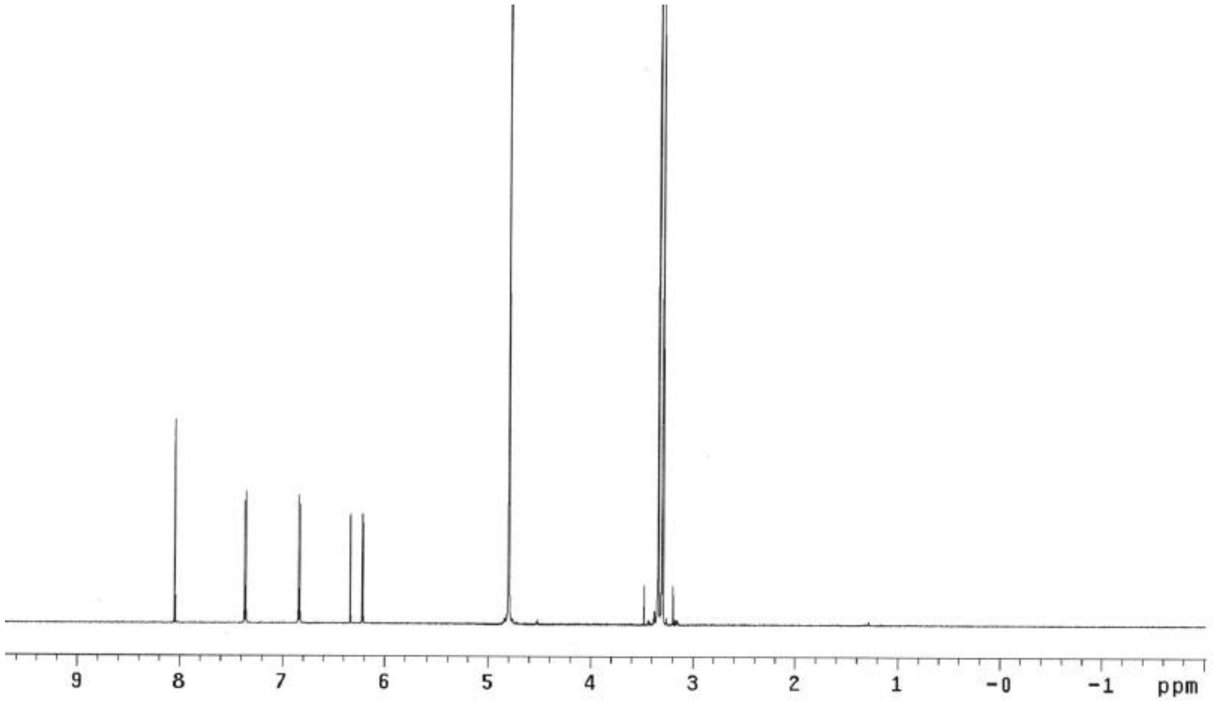

Figure S3 ¹H-NMR spectrum of genistein (compound 1) (CD_3OD; 500 MHz)

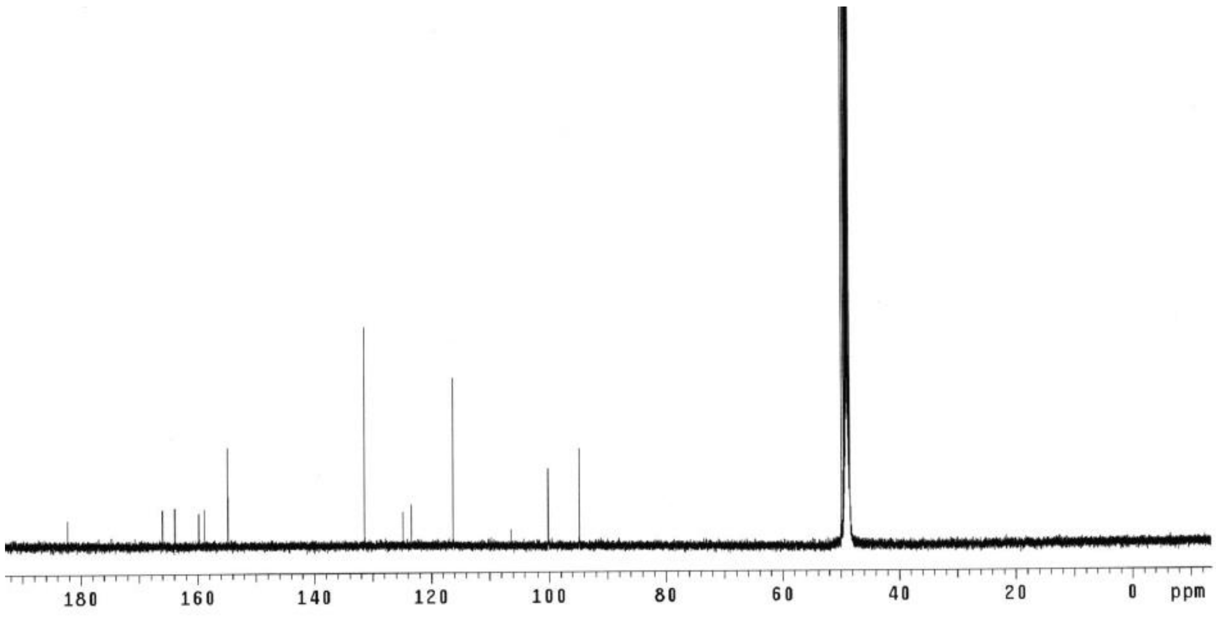

Figure S4 ¹³C-NMR spectrum of genistein (compound 1) (CD_3OD; 125 MHz)

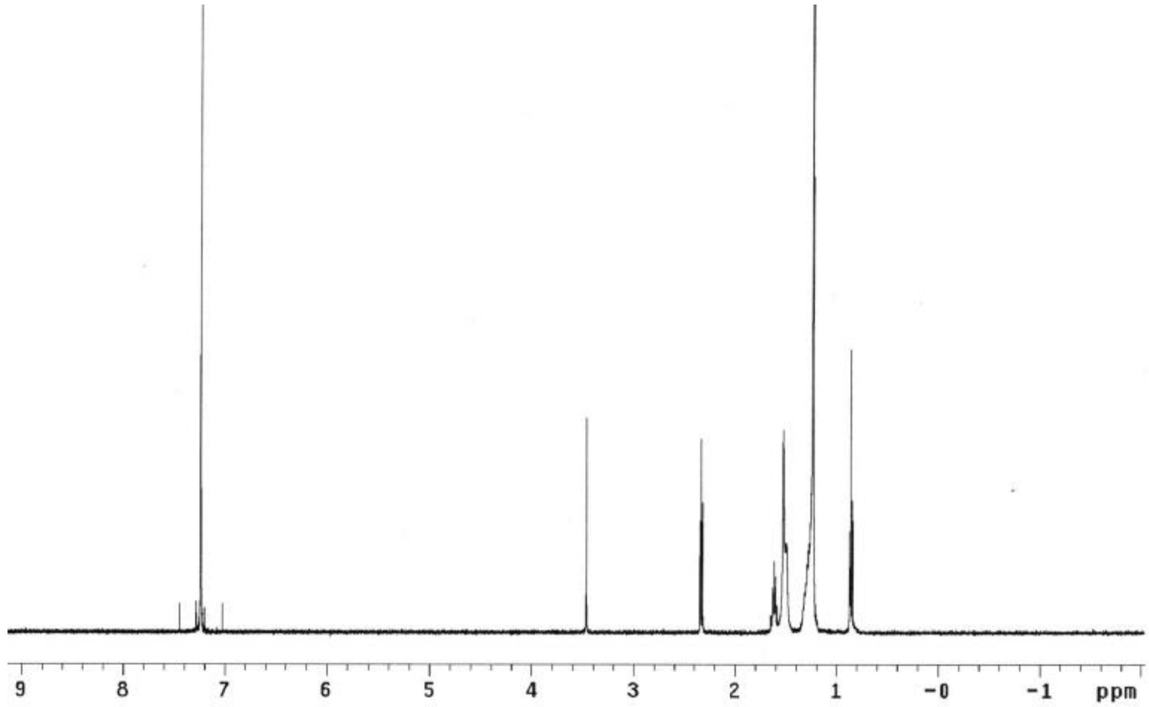

Figure S5 ^{1}H-NMR spectrum of hexadecanoic acid (compound 2) ($CDCl_3$; 500 MHz)

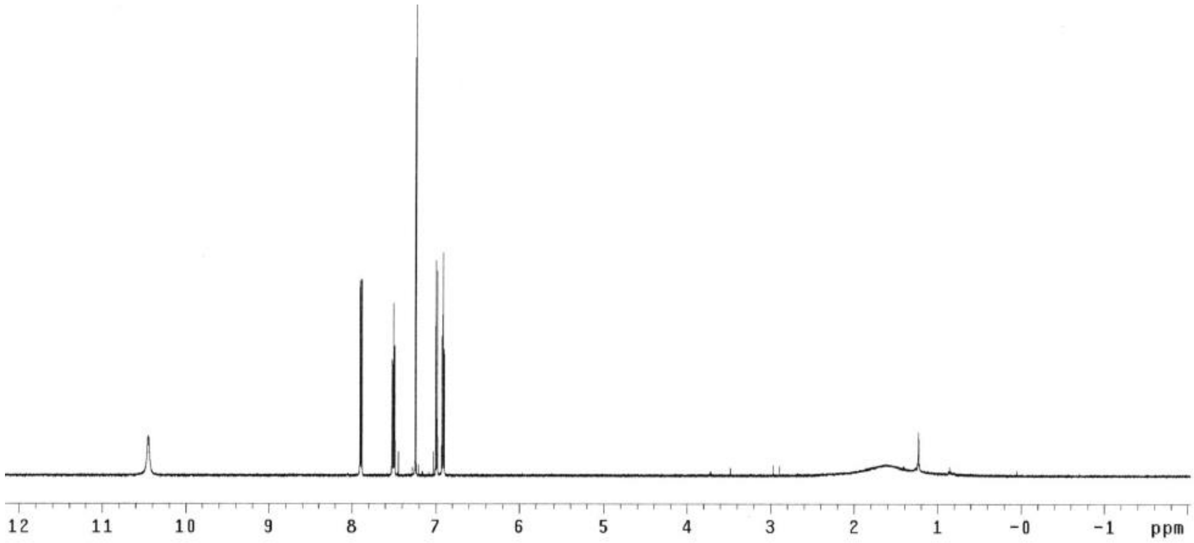

Figure S6 ^{1}H-NMR spectrum of salicylic acid (compound 3) ($CDCl_3$; 500 MHz)

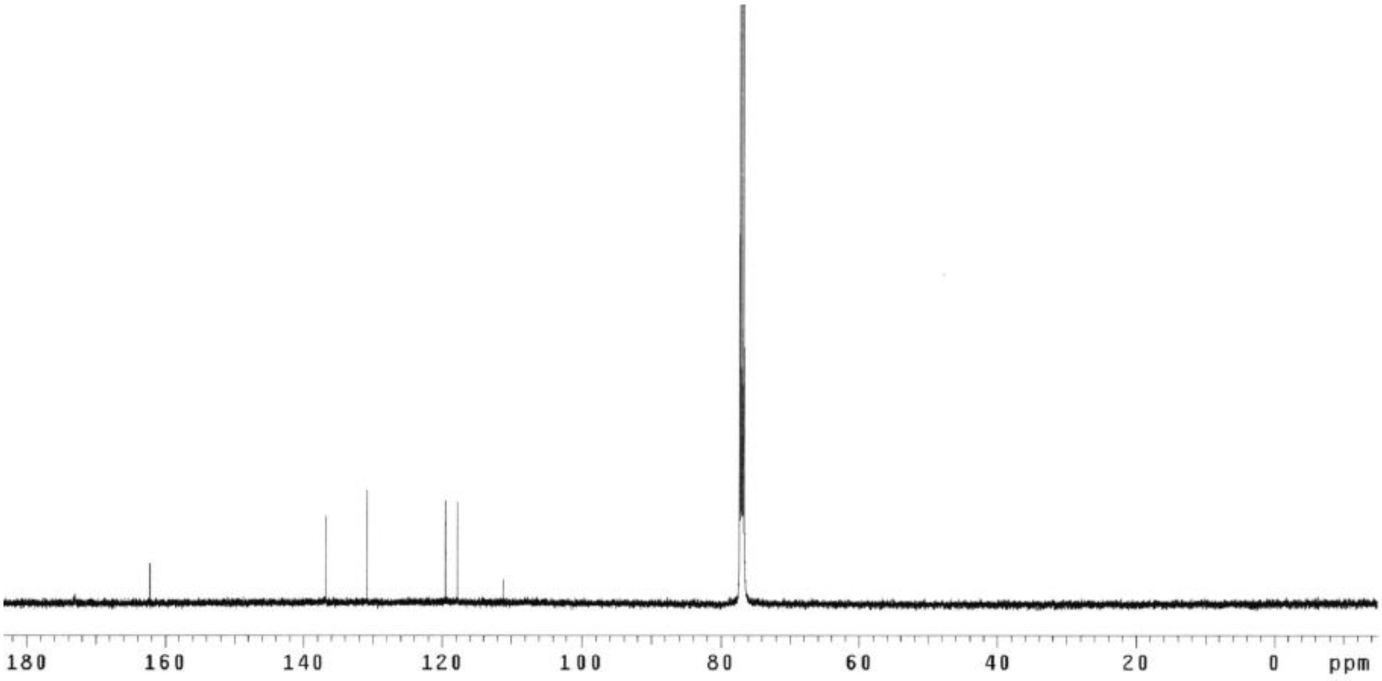

Figure S7 ^{13}C-NMR spectrum of salicylic acid (compound 3) ($CDCl_3$; 125 MHz)

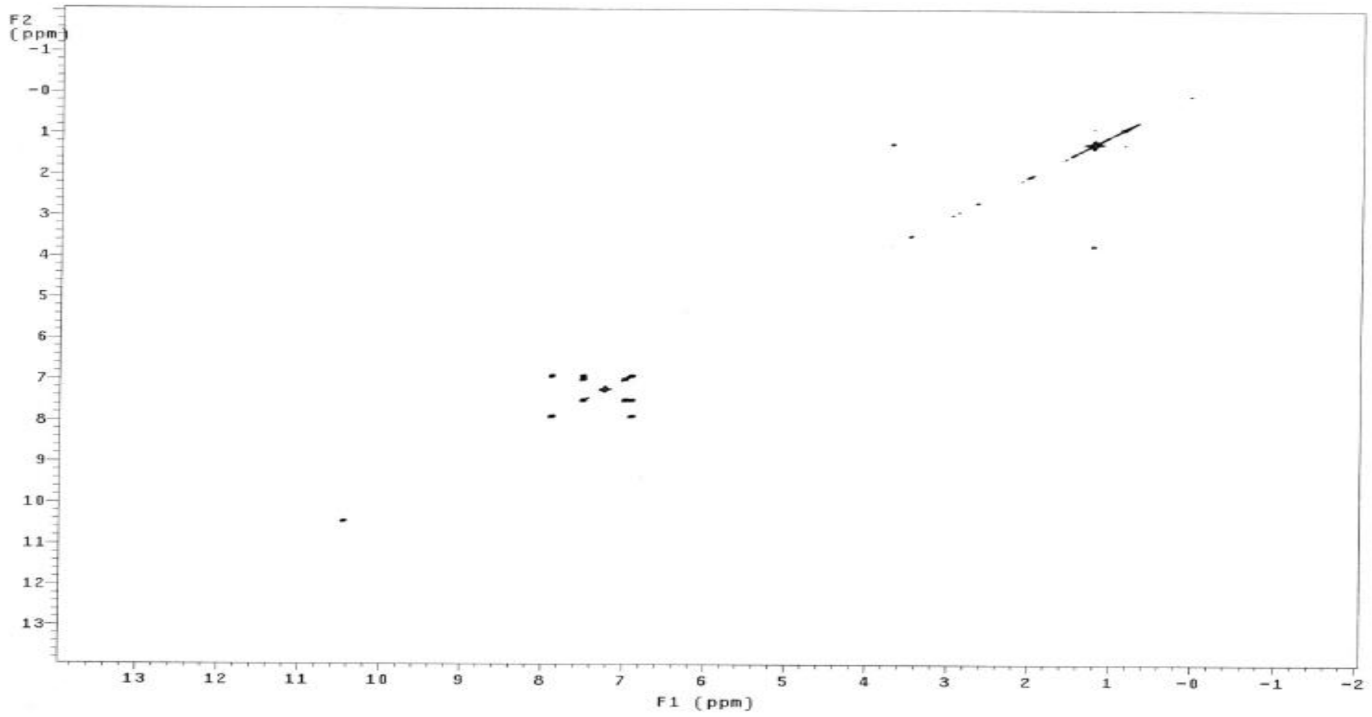

Figure S8 ^{1}H-^{1}H NMR spectrum of salicylic acid (compound 3) ($CDCl_3$)

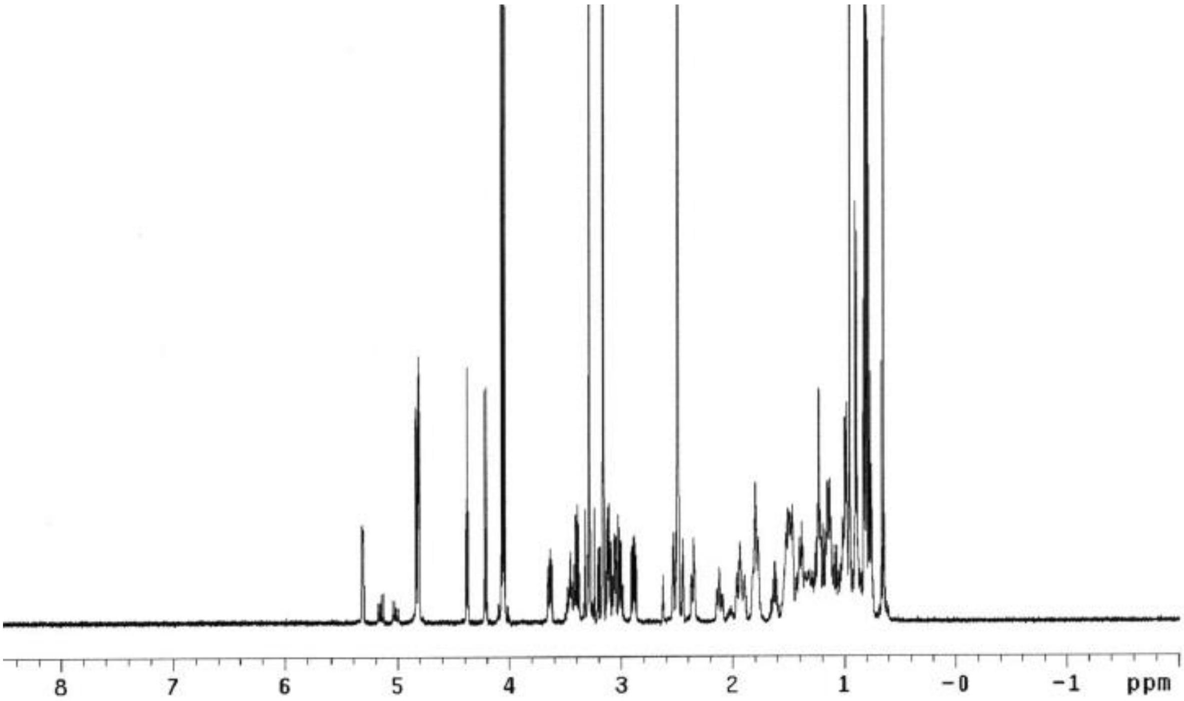

Figure S9 ^{1}H-NMR spectrum of β-sitosterol-D-glucoside (compound 4) (DMSO; 500 MHz)

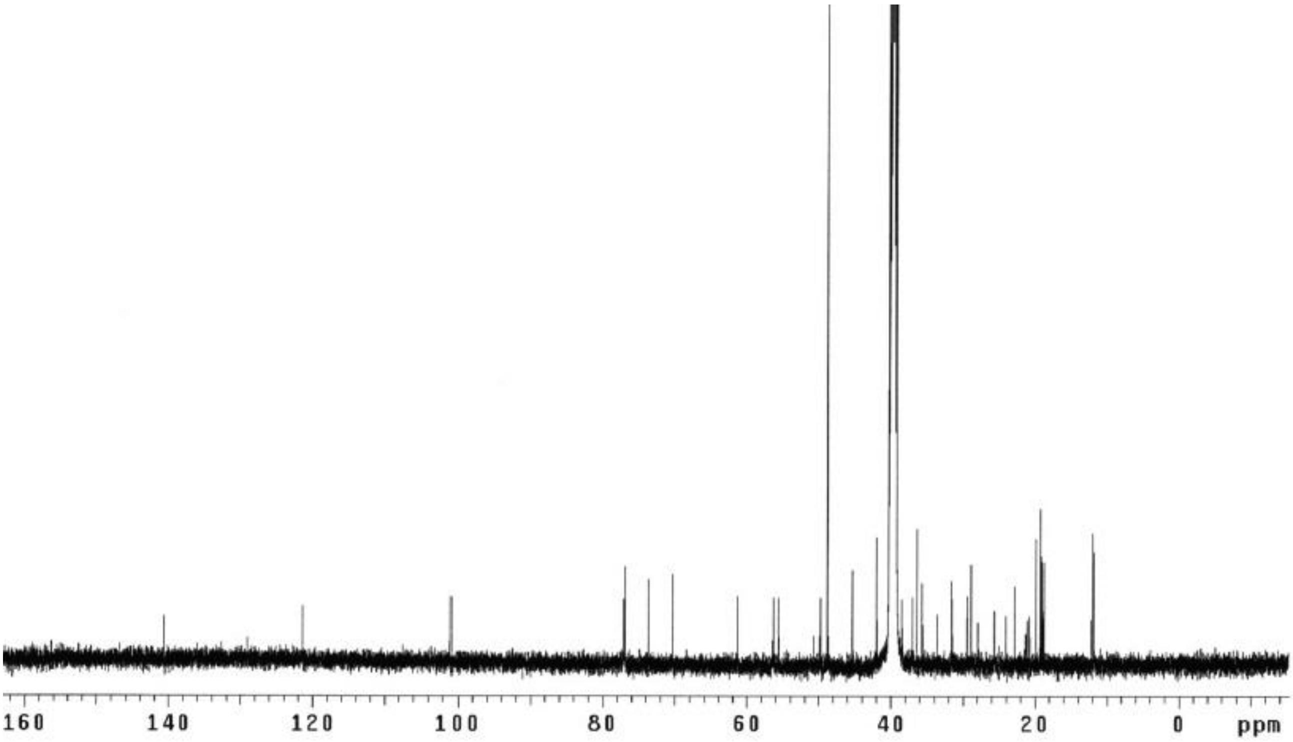

Figure S10 ^{13}C-NMR spectrum of β-sitosterol-D-glucoside (compound 4) (DMSO; 125 MHz)

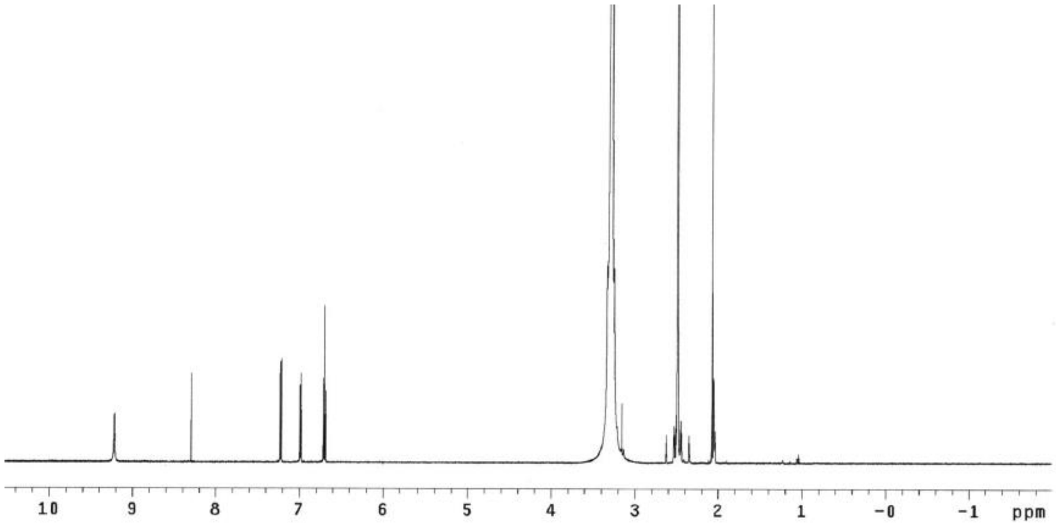

Figure S11 ^{1}H-NMR spectrum of 2,3-dihydroxybenzoic acid (compound 5) (DMSO; 500 MHz)

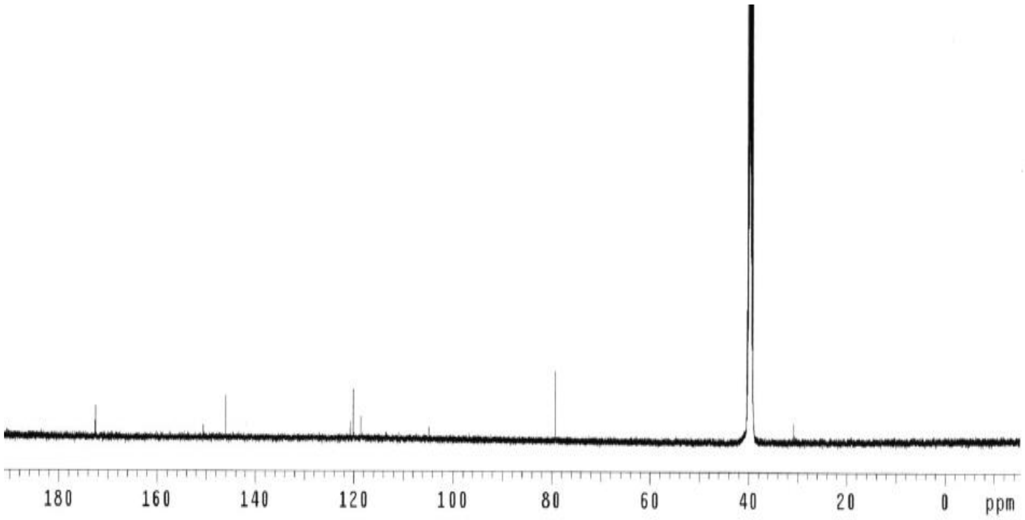

Figure S12 ^{13}C-NMR spectrum of 2,3-dihydroxybenzoic acid (compound 5) (DMSO; 125 MHz)

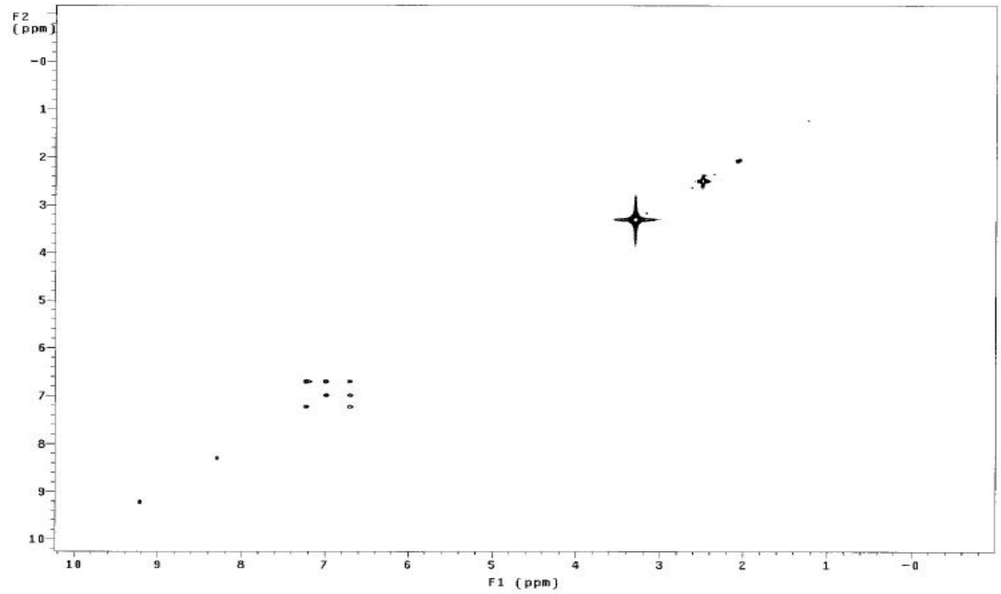

Figure S13 ^{1}H-^{1}H NMR spectrum of 2,3-dihydroxybenzoic acid (compound 5) (DMSO)

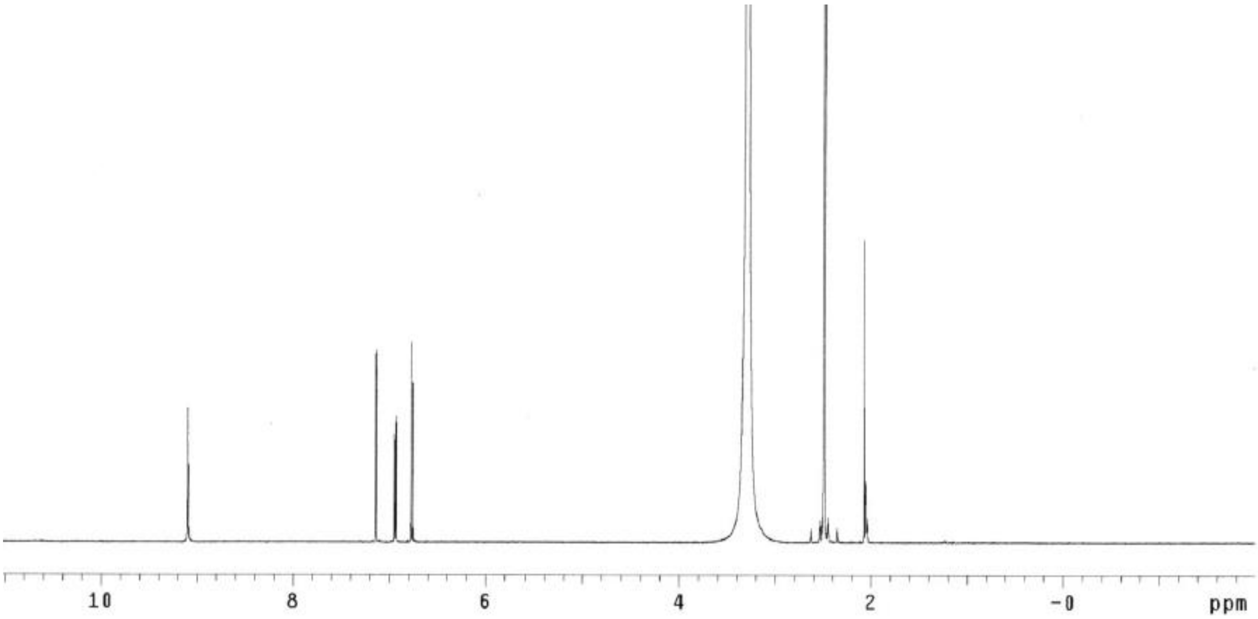

Figure S14 ^{1}H-NMR spectrum of 2,5-dihydroxybenzoic acid (compound 6) (DMSO; 500 MHz)

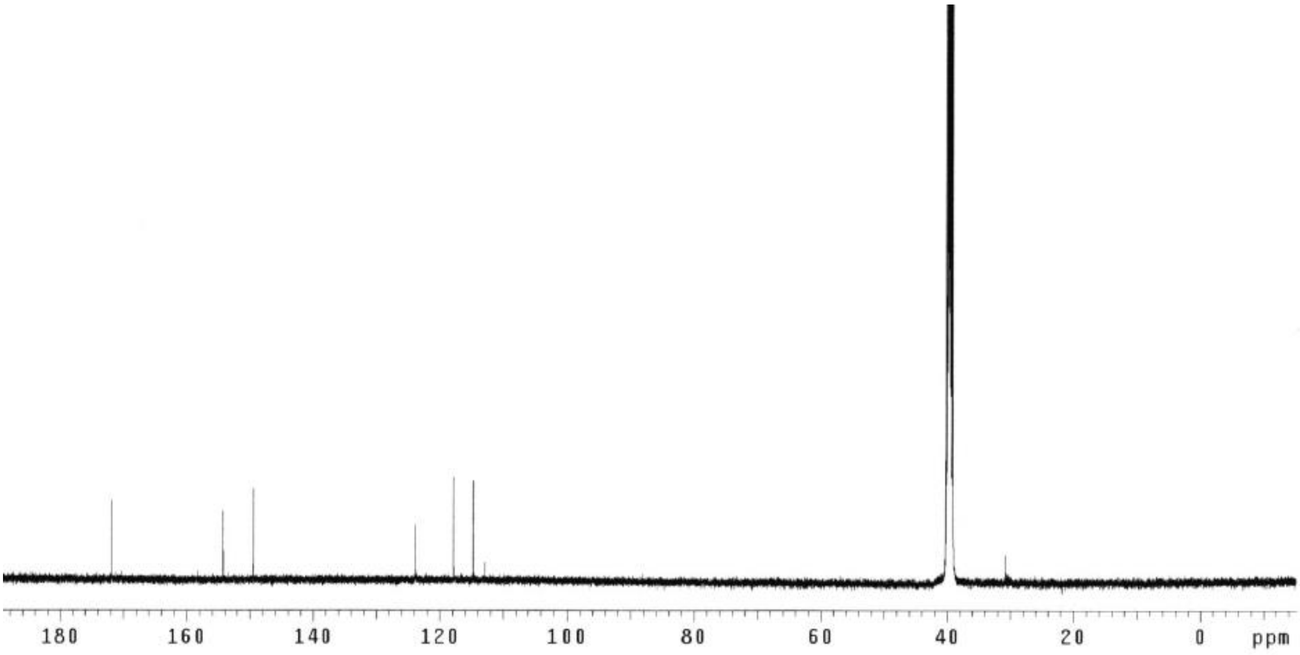

Figure S15 ^{13}C-NMR spectrum of 2,5-dihydroxybenzoic acid (compound 6) (DMSO; 125 MHz)

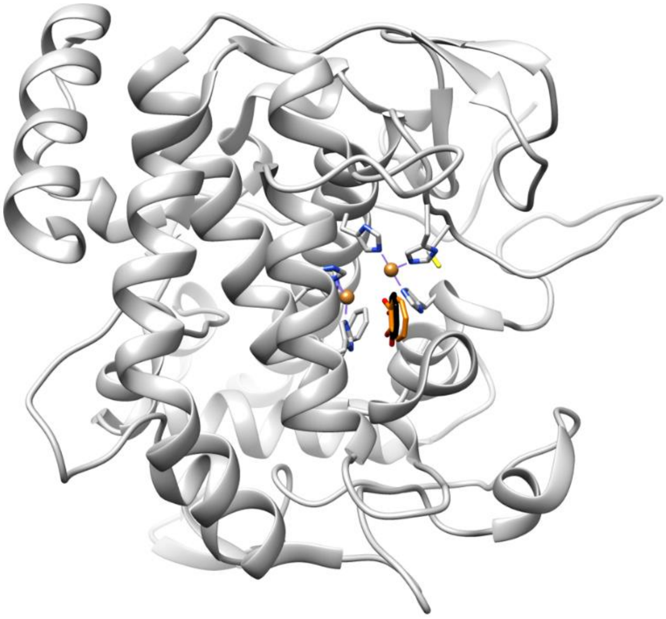

Figure S16. Docking validation via re-docking the extracted native ligand back (black) into its original position (organ). PDB ID: 2y9x is used as a target enzyme, fungal tyrosinase. The RMSD between the re-docked native ligand and its original pose is 1.070 Å.

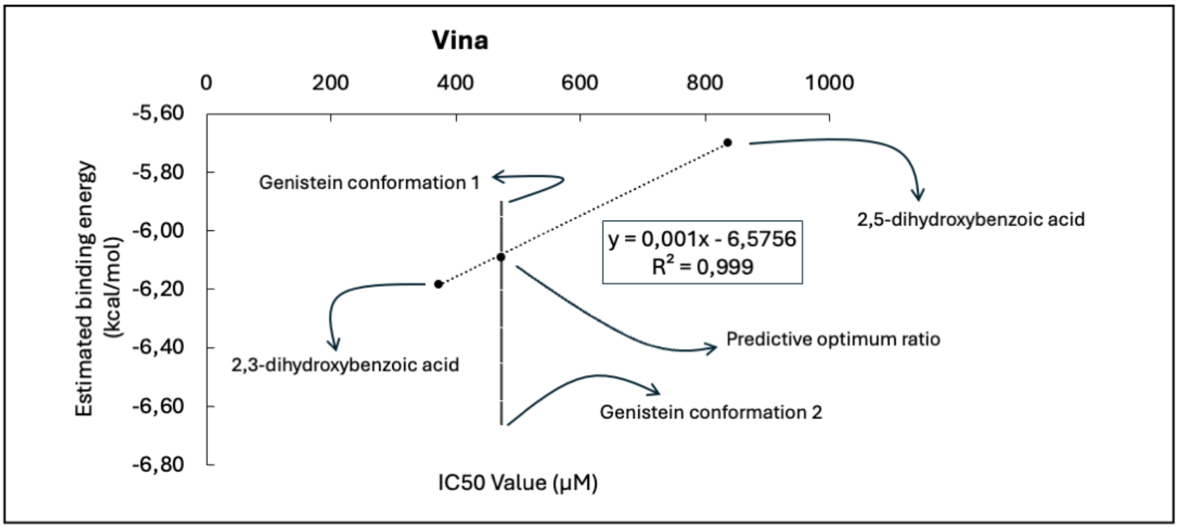

Figure S17. Predictive genistein conformation optimization guided by linear correlation between experimental data (IC_{50} value) and estimated binding energy from Autodock Vina.

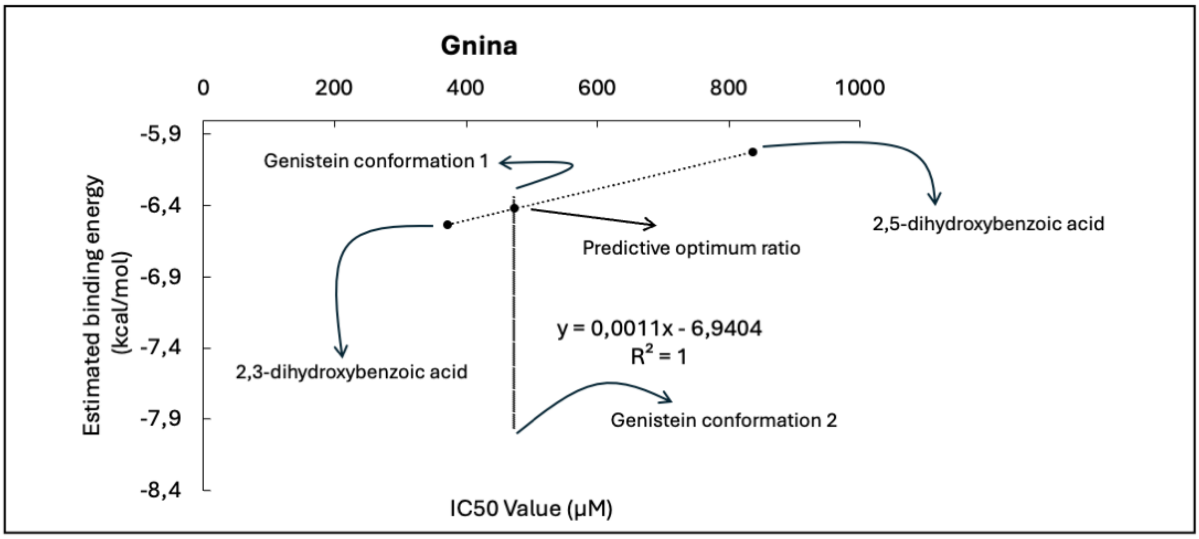

Figure S18. Predictive genistein conformation optimization guided by linear correlation between experimental data (IC50 value) and estimated binding energy from Gnina.
